# Forecasting the ecological footprint of G20 countries in the next 30 years

**DOI:** 10.1038/s41598-024-57994-z

**Published:** 2024-04-09

**Authors:** Rafael M. Eufrasio Espinosa, S. C. Lenny Koh

**Affiliations:** Advanced Resource Efficiency Centre, Management School, University of Sheffield, Sheffield, UK

**Keywords:** Environmental sciences, Sustainability

## Abstract

The Ecological Footprint evaluates the difference between the availability of renewable resources and the extent of human consumption of these resources. Over the past few decades, historical records have shown an accelerated decline in the availability of resources. Based on national footprint and biocapacity accounts, this analysis aims to advance the forecasting of the G20 countries' ecological footprints over a 30-year time frame. We employed a time series forecasting approach implemented in Python, which included-modular regression (Prophet) and Autoregressive Integrated Moving Average (ARIMA & Auto-ARIMA) methods. We evaluated and combined the performance of these three methods. The results indicated that among the largest economies of the G20, only four countries are projected to have a positive ecological footprint balance by 2050. These countries share the common denominator of large land areas and a moderate population growth projection. However, the overall trend of the indicator suggests that it will continue to decline.

## Introduction

One of the main objectives of the United Nations Sustainable Development Goal (SDG) 12, which focuses on responsible production and consumption for sustainable growth, is to mitigate the environmental degradation associated with population and economic growth, thereby facilitating a transition to a greener and more socially inclusive global economy^1,2^. However, this transition towards reducing our ecological footprint may be compromised, as all countries tend to prioritise economic growth over implementing mitigation actions^3,4^. Due to policies favouring economic growth and the advent of cheaper and more optimised energy sources, the demand for natural resources and their subsequent processing has dramatically increased since the last century, consequently enlarging our ecological footprint^5–7^. In some instances, this has caused severe harm to humankind^8^. Since 1961, humanity’s global ecological footprint has doubled, and currently, we consume renewable resources 20 to 50% faster than the planet can renew them^9^. Globally, no country has achieved sustainable resource use; in fact, we are moving further away from sustainable principles^10,11^. The overshoot of biophysical boundaries is set to continue escalating, and it is estimated that within just a few decades, the Earth's resource capacity will need to nearly double to keep pace with the current rate of population growth, production, and consumption^12^.

A few years ago, to grasp the extent of environmental challenges, an indicator developed by Wackernagel and Rees gained attention for its ability to measure and assess the sustainability of ecosystems. This gave rise to the concept of the ecological footprint (EF)^13^. Essentially, the EF aims to quantify the consumption of natural resources and the extent to which this consumption can be replenished by nature^14^. Within the EF terminology, ‘biocapacity’ refers to the capability of natural environments to regenerate the land surfaces utilised by humans^3^. The EF is considered an appropriate environmental quality index because it includes land use for crops, grazing, forests, fishing grounds, built-up areas, and the carbon footprint. It also sheds light on how economic activities affect the environment, both directly and indirectly^15^. Consequently, for many researchers, the EF emerges as a critical indicator, surpassing others that focus solely on specific environmental concerns like air pollution, carbon emissions, or global warming^16^. Historical records of this measurement system, dating back to 1961, are updated annually^17^. These records reveal that problems related to the global environmental footprint are intensifying, posing an increasing concern among managers, economists, and environmentalists. This concern is particularly pronounced in the G20 countries^18,19^.

The G20 consists of the world’s most influential economies, a combination of developed and developing nations, representing about two-thirds of the global population, 85% of the world’s gross domestic product, and over 75% of global trade^15^. Therefore, should the G20 nations achieve consensus on policies to mitigate the EF, the group could serve as an ideal platform for addressing these environmental issues, with the potential for these strategies to be adopted by other nations thereafter^16^.

Initially, ecological footprint (EF) forecasting faced criticism for its static analytical approach, primarily due to the high sensitivity in estimation methods^20^. However, prediction models are now increasingly recognised as vital instruments for policy purposes, particularly in developing mitigation scenarios. These models extend beyond the original EF methodology’s static approach by incorporating dynamic elements. Notably, country-level predictive models, based on multiregional input–output analysis, suggest that it is improbable for the world to avoid exhausting its bio productive capacity for human purposes by the century’s end^21^. In line with advancements across various research domains, the adoption of more sophisticated prediction techniques, such as machine learning from Artificial Intelligence (AI), is gaining momentum in EF forecasting. The advanced methods offer new insights into pattern recognition and environmental change prediction^22^.

The relevance of applying these innovative approaches within the G20 has been recognised, albeit with only a few examples to date. Given the pressing need to address the anticipated increase in consumption behaviours and the escalating negative footprint in these countries, there is a call for more research in this area^18,22,23^. In response, we have designed this resource footprint balance analysis within a forecasting framework, aiming to support planning for natural resource management. This approach is based on historical national footprint accounts provided by the Global Footprint Network (Figure SI 1)^13,17^.

The primary objective of this research is to predict the ecological footprint over the next 30 years, thereby facilitating improved protection of the ecosystems in the G20 countries. This involves leveraging new prediction techniques in time series modelling, including modular regression (Prophet) and autoregressive integrated moving average (ARIMA) methods.

The value of using time series forecasting for the ecological footprints of G20 nations lies in its ability to provide predictive insights based on historical data, guiding informed and timely decision-making for environmental sustainability. The significance of this approach is its role in enhancing data-driven policy making, allowing governments and organizations to anticipate future trends, allocate resources more efficiently, and implement targeted interventions to mitigate ecological impacts. The innovation in this methodology is in its application of advanced statistical and analytical techniques to environmental data, offering a new perspective on managing ecological footprints through predictive modelling and forecasting.

## Results

### Ecological footprint per capita

Projecting the ecological footprint in terms of global hectares per capita within the G20 countries is relevant for sustainability assessments, comparative analysis, policy formulation, climate change mitigation efforts, and fostering global cooperation. It offers essential insights into the environmental impact of these nations, aiding and guiding towards a more sustainable future by addressing potential resources scarcity issues.

**Figure 1:**
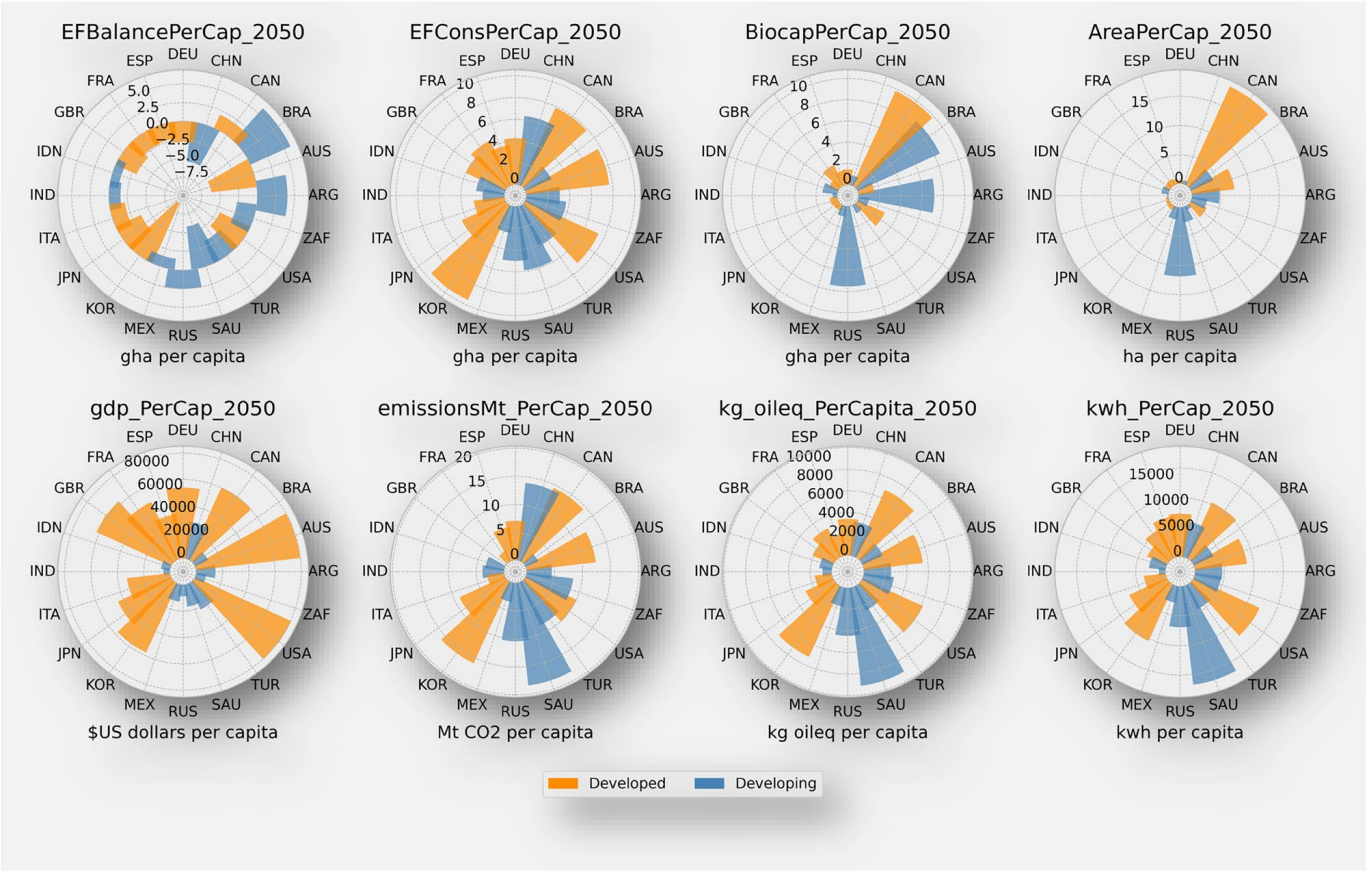
Plot shows a group of polar charts arranged from top left to bottom right displaying: The ecological footprint balance in global hectares per capita (gha/cap) by the 2050 circa year where developed countries are illustrated in orange colour and developing countries in blue, the ecological footprint consumption (gha/cap), biocapacity per capita (gha/cap), total area per capita (gha/cap), GDP per capita in US dollars, Emissions per capita in MtCO_2_, Fuel consumption per capita in kg Oil-eq, and electricity consumption per capita in kWh.

This analysis investigates key variables such as balance per capita, consumption per capita, biocapacity per capita, area per capita, GDP per capita, electricity use per capita, emissions per capita, and fossil fuel consumption per capita. These variables help understand the patterns and trends of each country's ecological footprint (Fig. 1). The G20 countries are categorised into developed and developing groups for this analysis. The developed category includes Australia (AUS), Canada (CAN), Germany (DEU), Spain (ESP), France (FRA), the United Kingdom (GBR), Italy (ITA), Japan (JPN), South Korea (KOR), and the United States (USA). The developing category comprises Argentina (ARG), Brazil (BRA), China (CHN), Indonesia (IDN), India (IND), Mexico (MEX), Russia (RUS), Saudi Arabia (SAU), Turkey (TUR), and South Africa (ZAF).

The Ecological Footprint (EF) Balance per capita assesses the ecological footprint on a per-person basis, revealing whether a country's consumption of resources exceeds or aligns with its biocapacity. Developing countries, notably Argentina and Brazil, demonstrate positive balances per capita, suggesting their ecosystems could sustainably provide for their future demand for resources. This may be attributed to a variety of factors, including lower levels of population growth, industrial development, or less intensive use of resources compared to developed countries.

On the other hand, except for Canada, developed countries like Australia, the USA, Germany, and others exhibit negative balances per capita, indicating that their resource consumption exceeds their biocapacity.

EF Consumption per capita quantifies the average resource consumption by an individual in a country. Typically, developed countries display higher consumption levels per capita due to higher living standards and greater resource accessibility. For instance Australia, Canada, and Germany are among the countries with relatively higher consumption per capita, mirroring their resource-intensive economies and affluent lifestyles. Conversely, developing countries like China, India, and Mexico show lower consumption per capita, likely due to lower income levels, restricted resource access, and different consumption habits.

Biocapacity per capita measures the availability of biologically productive land and water resources per person. Developed countries, especially those with vast land areas and low population densities, such as Australia and Canada, exhibit higher biocapacity per capita. These countries possess abundant natural resources, including forests, agricultural land, and water bodies, which contribute to their higher biocapacity. In contrast, countries with high population densities or smaller land areas and limited access to resources, such as China, India, and South Korea, demonstrate relatively lower biocapacity per capita.

Area per capita reflects the land area available to each individual within a country. Countries with larger land masses, such as Canada, Australia, and Russia, exhibit higher area per capita, suggesting greater availability of natural resources and potential for ecosystem services. Conversely, countries with high density population or small land areas, such as China, India, and South Korea, have lower area per capita. This may pose challenges in managing resource demands and environmental conservation within limited spaces.

**Figure 2:**
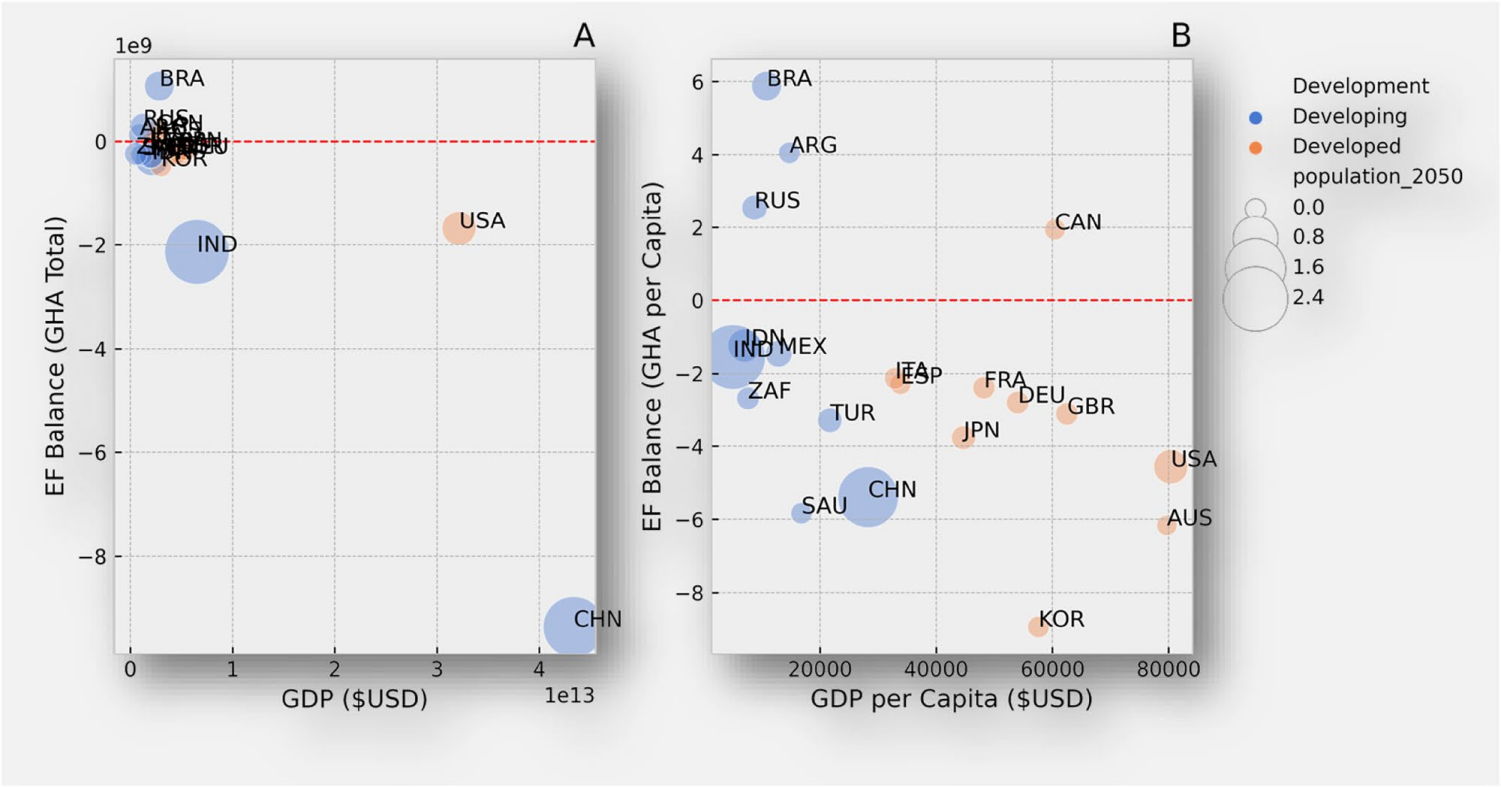
**(A)** Depicts the relationship between forecasted GDP in US dollars and Ecological Footprint in total global hectare for each country by the year 2050. The size of the bubbles corresponds to the projected population size in billions, with blue dots representing developing countries and orange dots representing developed countries. (**B)** Illustrates the relationship between forecasted GDP per capita and Ecological Footprint per capita for each country by the year 2050. Similar to the previous plot, the size of the bubbles corresponds to projected population size in billions, with blue dots indicating developing countries and orange dots indicating developed countries.

GDP per capita represents the economic output per person and serves as a proxy for the overall economic development of a country. Countries characterized by higher GDP per capita, such as the United States, Australia, the United Kingdom, and Canada, exhibit stronger economies, technological advancements and higher standards of living. In contrast some developing countries, despite having high potential in terms of natural resources, skilled labour, or other assets, may still have a low GDP per capita. For example, emergent economies in Asia such as India, Indonesia, or in the Americas like Brazil and Mexico will continue to face challenges related to income inequality, political stability, lack of infrastructure, and investment, among other factors. The correlation between higher GDP per capita and ecological footprint is complex. While some developed countries have successfully decoupled economic growth from environmental degradation through sustainable practices, others continue to face challenges in achieving sustainable development (Fig. 2).

Electricity per capita measures the average electricity consumption in kWh per person, reflecting the energy demands and infrastructure development within a country. Overall Developed countries, with higher industrialization and urbanization rates, typically exhibit higher electricity consumption per capita. The United States, South Korea, Canada, and Australia are among the countries with relatively higher electricity consumption per capita, attributable to factors such as larger populations, energy-intensive industries, and higher standards of living that require more electricity for residential, commercial, and industrial purposes. While the highest electricity consumption among the G20s is found in Saudia Arabia, developing countries generally have lower consumption projections.

Emissions per capita indicates the amount of carbon dioxide (CO_2_) emissions per person, serving as a measure of a country's contribution to global greenhouse gas emissions. Developed countries, due to higher levels of industrial activity and energy consumption, generally exhibit higher emissions per capita. Countries like South Korea, Canada, Australia, and the United States have higher emissions per capita. Among developing countries, Saudi Arabia has higher emissions per capita, while the United Kingdom is projected to be the only nation with negative emissions by 2050.

Fossil fuels per capita represents the amount of fossil fuels consumed per person within a country. Developed countries, which often rely heavily on fossil fuel-based energy sources, typically exhibit higher fossil fuel consumption per capita. South Korea, Canada, and the United States have relatively higher consumption. Among developing countries, Saudi Arabia, shows the highest consumption in kg oil equivalent per capita. This underscores the need for a transition to cleaner and more sustainable energy sources to reduce dependency on fossil fuels and mitigate climate change impacts. Countries with lower consumption of fossil fuels, like India and Indonesia, have large populations, which might result in per capita consumption appearing lower even if overall consumption increases.

### The G20 in 2050

Figure 2 illustrates the importance of comparing the total ecological footprint of countries with their per capita footprint per capita, offering a comprehensive perspective on sustainability within the G20 group. Assessing countries’ total ecological footprints alone, as shown in Fig. 2A, it can sometimes mask disparities. For instance, a country might appear unsustainable based on its large total footprint; however, a high population by per capita footprint is relatively smaller. Conversely, a country with a small total footprint might appear sustainable, but if wealth and consumption are concentrated among a small population, its per capita footprint could be significantly higher. Comparing per capita footprints (Fig. 2B) allows for a more equitable evaluation of environmental responsibility. The United Nations Sustainable Development Goals (SDGs) stress the necessity of balancing economic growth with environmental protection for sustainable development. Thus, analyzing ecological footprints per capita is essential for highlighting the importance of harmonizing advancement in living standards with efforts to reduce environmental impact.

**Figure 3:**
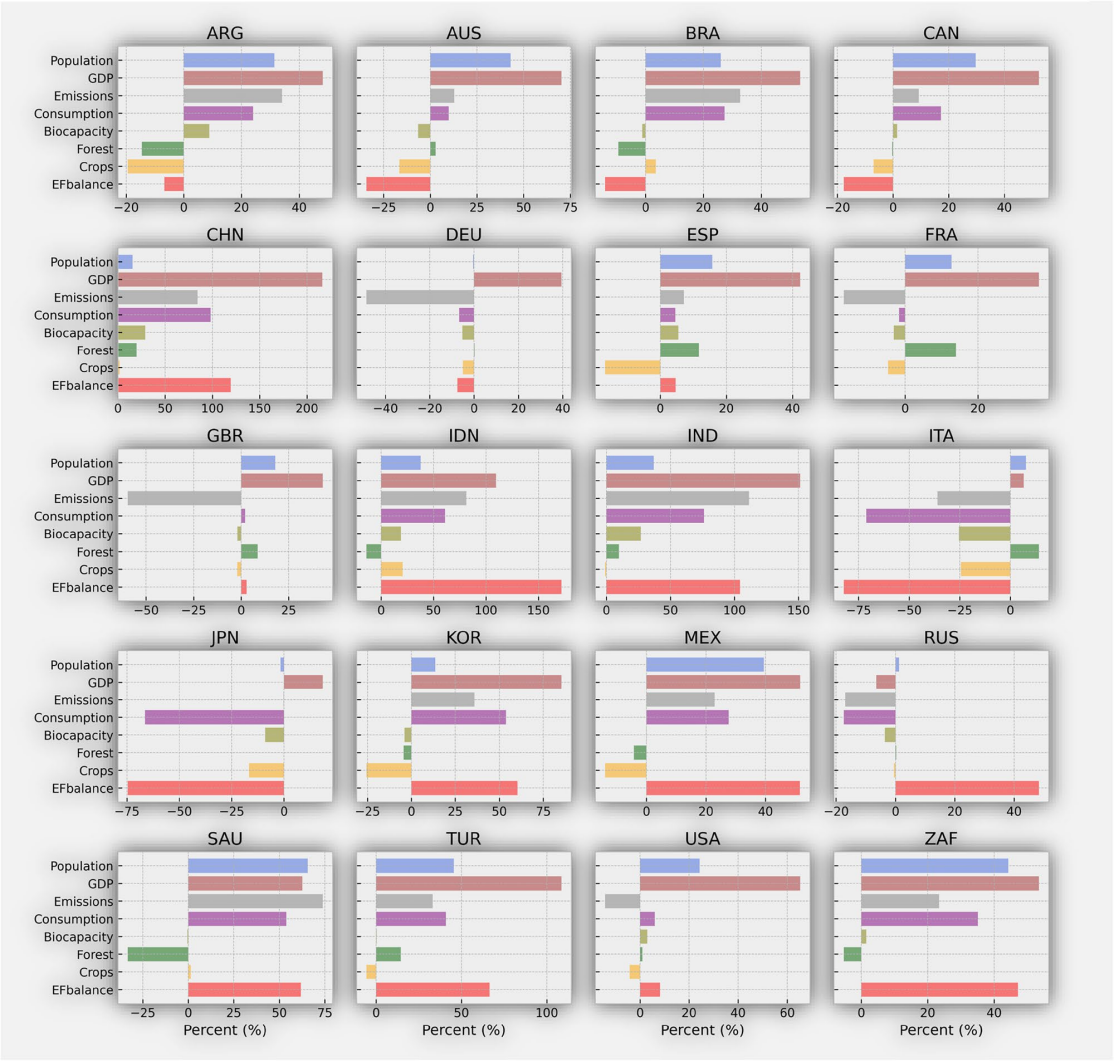
Plot shows the forecast change in percent at country level between 2018 and 2050, for the following variables: Population in blue color, GDP in brown, Emissions in gray, Consumption in purple, Biocapacity in olive, Forest area in green, Cropland area in yellow and the ecological footprint balance in red. ARG = Argentina, AUS = Australia, BRA = Brazil, CAN = CAN, CHN = China, DEU = Germany, ESP = Spain, FRA = France, GBR = United Kingdom, IDN = Indonesia, IND = India, ITA = Italy, JPN = Japan, KOR = South Korea, MEX = Mexico, RUS = Russia, SAU = Saudi Arabia, TUR = Turkey, USA = The United States, and ZAF = South Africa.

Figure 3 presents the forecasted percentage change between 2018 and2050 for the main drivers affecting the total ecological balance of each nation (refer to Figure SI 24 for forecasted values). Developing countries, particularly in Asia and Africa, are anticipated to see significant population growth by 2050. Turkey, Saudi Arabia, and Indonesia are expected to experience high population growth rates of 45.47%, 65.59%, and 37.89% respectively. In contrast, developed countries such as Germany, Italy, and Japan are projected to have slower, or even negative population growth rates (Figure SI 2–4). Economically, developed countries are generally expected to exhibit higher GDP growth rates compared to their developing counterparts. Australia, Canada, and the United States have significant projected GDP growth rates of 70.25%, 52.40%, and 65.27%, respectively. Among developing countries, China stands out with an extraordinary projected GDP growth rate of 216.22%. Other developing nations like India (151.29%), Indonesia (109.625), Turkey (108.58%), and Brazil (53.51%) are also forecasted to show considerable GDP growth rates, as shown in Figure SI 5–7.

Between 2018 and 2050, emissions in developing countries, particularly China and India, are projected to witness significant increases, with forecasts showing rises of 84.34% and 111.36% respectively. This trend underscores the rapid economic development and large populations bases in both countries, which fuel increased energy demand, industrial activities, and consequently, emissions. Without interventions like enhanced energy efficiency or a shift towards cleaner energy sources, emissions are expected to grow substantially. Conversely, developed countries such as Germany, France, and the United Kingdom are projected to see reduction in emissions during the same period (Figure SI 8–10). These nations often implement more stringent environmental regulations, face greater public and international pressure for emission reductions, and possess the resources necessary for enforcing these regulations. As leaders in the transition to a low-carbon energy system, these countries demonstrate the potential for sustainable growth alongside emissions mitigation.

Developing countries such as China, India, and Indonesia are forecasted to experience significant increases in consumption growth rates, projected at 97.96%, 76.24%, and 61.06% respectively. These increases are largely driven by rapid economic development, which elevates income levels and consumer capacity for goods and services. Such economic growth typically involves resource-intensive industrial activity and construction, contributing to higher consumption levels.

In contrast, developed countries like Germany, France, and Japan are projected to see reductions in consumption (Figure SI 11–13). Factors such as lower population growth rates or aging populations in these nations may contribute to decrease consumption levels, as a smaller, older population typically demands fewer resources. Overall, the differences in projected consumption growth rates between developing and developed countries can be attributed to a variety of factors related to economic growth, demographic changes, and policy measures among others.

Developed countries, including Germany, France, Italy, and Japan are expected to face ecological deficits, with projections indicating negative biocapacity growth rates. These suggest these nations may struggle to sustain their ecological resources relative to consumption (Figure SI 14–16). Conversely, developing countries such as Brazil, China and Indonesia show mixed projections for biocapacity, with both increases and decreases anticipated, indicating varied impacts of development and conservation policies on their ecological capacities.

The projections for forest area also differ significantly between developed and developing countries. Countries like Australia, Germany, and Italy have negative forestry area projections, implying initiatives towards reforestation and possibly a reduction in deforestation rates. On the other hand, developing countries including Brazil, Indonesia, and Turkey are projected to see positive changes in forest areas, suggesting potential expansion of reforestation activities (Figure SI 17–19).

In terms of agriculture, developing countries, including Brazil, Indonesia, and Turkey anticipate increases in crop production, reflecting expected growth in agricultural output. This growth is likely driven by improvements in agricultural practices and expansion in agricultural lands. Developed countries such as Germany, France, and Italy present mixed projections for crop production. These variations may reflect the impact of technological advancement policy measures, and environmental considerations in agriculture (Figure SI 20–22).

The Ecological Footprint Balance offers insights into the sustainability and resource management of countries. Typically, developed countries display negative balances, showing that their resource consumption and waste production exceed their ecological capacity. Specifically, Italy, Japan, and Australia are projected to have significantly large negative ecological footprint balances by 2050 compared to their levels in2018. In contrast, some developing countries like Indonesia, India, and Turkey are expected to exhibit positive ecological footprint balances, indicating a more sustainable resource use.

Regionally, Asia, particularly China and India, stands out in terms of population, GDP growth, emissions, and consumption, due to their large populations and fast-growing economies. These factors substantially influence global trends. Africa, with South Africa as an example, experiences high population growth rates. In Europe, developed countries like Germany, Italy, France, and the United Kingdom show varied projections across different indicators, reflecting diverse economic and sustainable strategies.

This analysis underscores the critical need for tailored sustainability and resource management practices across different countries and regions to address the challenges posed by varying economic developments and ecological capacities.

### Historical analysis

**Figure 4:**
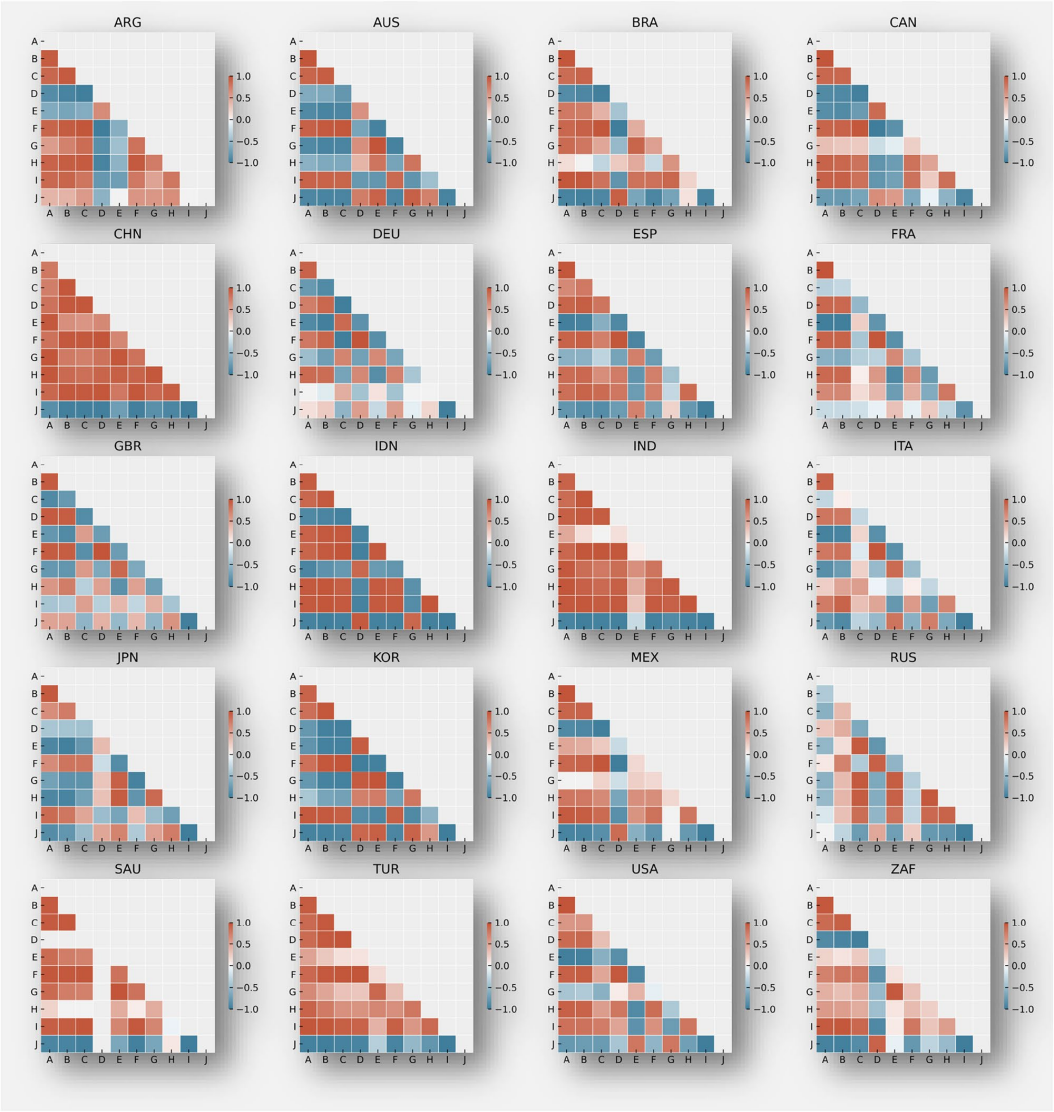
Plot shows a country-by-country historical correlation charts where: A = 'population', B = 'gdp', C = 'emissions_kt', D = 'forest_km2', E = 'crops_km2', F = 'HDI', G = 'AreaTotHA', H = 'BiocapTotGHA', I = 'EFConsTotGHA', J = 'deficitTotGHA' . Correlation values range from -1 to 1 and indicate the strength and direction of a linear relationship between the variables. A correlation between 0 and 1 indicates a positive linear relationship where the strength of the relationship increases with the value. A correlation between 0 and − 1 indicates a negative linear relationship where the strength of the relationship increases with the value (in absolute terms). ARG = Argentina, AUS = Australia, BRA = Brazil, CAN = CAN, CHN = China, DEU = Germany, ESP = Spain, FRA = France, GBR = United Kingdom, IDN = Indonesia, IND = India, ITA = Italy, JPN = Japan, KOR = South Korea, MEX = Mexico, RUS = Russia, SAU = Saudi Arabia, TUR = Turkey, USA = The United States, and ZAF = South Africa.

Historical analysis of relationships between various indicators in developed and developing countries uncovers significant patterns (Fig. 4), emphasising that while these correlations reveal insightful trends over time, they do not imply causation.

In developed countries like the United States, Germany, and Japan, key historical patterns emerge. A consistent positive correlation between population and GDP indicates that economic growth has often accompanied population increases, likely due to an expanding labour force and market size. Similarly, a positive correlation between GDP and emissions suggests that economic expansion has typically been linked with increased industrial activity, energy consumption and higher emissions. Conversely, emissions and forest area have a negative historical correlation, indicating that rising emissions are associated with decreasing forest areas, reflecting the impact of industrialization and deforestation. Additionally, the Human Development Index (HDI) and forest area show a strong negative correlation, suggesting that advancements in human development have historically led to deforestation and land development (Figure SI 23).

For developing countries, including Argentina, Brazil, China, India, Indonesia, Mexico, and Turkey, distinct trends are observed. A strong historical positive correlation between population and GDP in these countries point to economic growth driven by an increasing population and consumer demand. The positive correlation between GDP and emissions indicates that economic growth has historically resulted in higher emissions due to escalated industrial activities and energy consumption. The relationship between emissions and forest area varies, with some nations showing positive correlations, possibly due to efforts in afforestation or natural reforestation over the years, while other cases show negative correlations, reflecting deforestation trends.

Additionally, historical correlations between HDI and emissions in several developing countries have shown a positive trend. This suggests that as the HDI has improved historically, emissions have tended to increase as well. This historical pattern indicates that higher levels of historical human development have been associated with increased energy consumption and industrial activities in these countries. Furthermore, in several developing countries, there has been a historical negative correlation between population and forest area. As the population has increased over time, the area covered by forests has tended to decrease, indicating the historical impact of human activities on deforestation and land-use change in these countries.

Comparing developed and developing countries: When comparing the historical correlations between developed and developing countries, some similarities and differences emerge. Both groups exhibit historical positive correlations between population and GDP, indicating the role of population growth in driving economic development. However, the historical correlations between emissions and forest area vary, highlighting the complex relationship between human development and forest conservation efforts in different contexts.

In summary, the historical relationships observed in developed and developing countries provide insights into the complex interplay between population, GDP, emissions, and forest area. Understanding these historical correlations can inform policymakers and stakeholders in developing strategies for sustainable development and environmental conservation. It is important to consider the unique circumstances and dynamics of each country while formulating effective policies and interventions to balance economic growth and environmental sustainability.

## Discussion

In this analysis, we forecast the ecological balance of the G20 countries over the next 30 years. The objective of the study was to glimpse the future environmental impact of these countries, which could be crucial for promoting sustainable development through responsible resource consumption, ensuring a healthier and more resilient planet for future generations. The research employed a combination of time-series forecasting models and data analysis techniques, incorporating data on population growth, economic development, and energy consumption patterns. We recognise that the membership of the G20 might change by 2050^24^, but for the purpose of this analysis, we retained the current members.

Our results indicated that developed countries exhibit negative ecological balances per capita, meaning their resource consumption has already exceeded biocapacity, while most developing countries show positive balances per capita, indicating a higher resource demand than what their ecosystems can sustainably provide. In this context, developed countries generally have higher consumption due to greater access to resources and higher living standards, whereas developing countries experience lower consumption due to lower income levels and limited resource access. Biocapacity per capita is proportional to the country’s extension, reflecting the land area available per individual, with larger countries like Canada, Australia, and Russia having more area per capita and potentially greater ecosystem resilience. Based on current trends, it is foreseeable that by 2050, only a few developed countries will have successfully decoupled economic growth from environmental degradation through the adoption of sustainable practices.

However, average electricity and fossil fuel consumption per person will likely remain higher in developed countries due to their higher levels of industrialisation and urbanisation, resulting in higher emissions per capita. Among developing nations, only three countries are projected to maintain a positive balance by 2050, while the deficit will continue to increase in the rest of the nations. Overall, these findings highlight the disparities between developed and developing countries in terms of ecological footprint, resource consumption, and environmental impact. They underscore the importance of adopting sustainable practices, transitioning to cleaner energy sources, and collectively addressing environmental challenges to ensure a more balanced and resilient planet.

Compared with other forecasting approaches, Lenzen and Wiedman's study^21^ on a multi-regional macroeconomic model concluded that the ecological footprint of countries will, on average, increase by approximately 30% by 2050, resulting in a reduction of biocapacity to only 0.8–0.9 gha/cap. This aligns with our projections for developed countries within the G20, indicating a clear trend of increasing ecological footprint. Another study^15^ suggests that while non-renewable energy consumption has negatively impacted environmental quality in G20 countries, globalization, renewable energy, and urbanization have contributed to environmental quality improvement. Here, we highlight that pathways to mitigate the ecological footprint through green energy sources will differ between developed and developing nations. It's essential to remember that each study has its methodology, data sources, and specific scope, so direct comparisons in forecasting studies may not always be straightforward but can help to identify common problems.

Regarding the forecasting methods employed in this study, we opted for a simple univariate analysis^25^, considering that a group of variables is already incorporated in the original calculation method from the global network footprint. Thus, we assume that the evolution of these variables is already reflected in the total footprint balance in each country. However, further investigation using a multivariate approach by considering the interrelations of variables for time series forecasting^26,27^ is recommended.

With the advancements in time series analysis and the utilization of big data, various methods, including Machine Learning, have been employed for forecasting. For our analysis, we combined traditional Autoregressive Integrated Moving Average (ARIMA) methods with AUTO-ARIMA and the modular regression method Prophet, developed by Facebook^28^. While ARIMA and AUTO-ARIMA specialise in utilizing historical data to generate future values, Prophet aims to identify changing points in trends. This combination of models has been used in previous studies across different research fields^29,30^.

In evaluating the individual methods performance, the root mean squared error (RMSE) showed some variations in the forecast results. Three methods performed moderately well and yielded similar outcomes, with ARIMA and AUTO-ARIMA demonstrating slightly better average performance than Prophet, although with minor differences. However, the mean absolute percent error (MAPE) in some datasets was lower in Prophet. The combined average methods for the evaluation forecast for each variable is shown in Tables SI 1–13.

Based on our analysis, the findings presented and integration with the forecasting of established environmental indicators from the OECD, we propose the following general recommendations. These suggestions are intended to inform future research directions, policy-making, or practical applications in the field. It is important to note that while these recommendations are derived from our current study, their applicability and effectiveness should be considered in the context of specific circumstances and validated through further empirical research.

The Environmental Policy Stringency Index (EPS)^31^, indicates that countries like France and Germany, with their high stringency scores and upward trends, should keep advancing their regulatory frameworks. In contrast, nations such as Russia and South Africa, which exhibit lower or inconsistent EPS scores, would benefit from enacting more robust policies and leveraging international partnerships to bolster their environmental policy infrastructures. It's crucial for these developing countries to establish regulations that support sustainable development and a shift toward eco-friendly economies (Figure SI-24).

Further analysis of international environmental policies reveals the diverse phases of policy maturity among countries^32,33^. The UK and France, characterized by strong and progressively stringent policies, should persist in refining these strategies and nurturing investments in green technology. On the other hand, nations like India and Indonesia, which show less stringent policy landscapes, should concentrate on reinforcing their policy frameworks and magnifying the scale of current strategies. This includes formulating incentives for renewable energy adoption, enhancing energy efficiency, and setting tougher emissions regulations. International support could play a pivotal role in upgrading their policy tools and infrastructures for effective climate change mitigation (Figure SI-26).

For developed nations, the path forward involves intensifying the integration of climate policies across various sectors to ensure a comprehensive reduction in emissions^32,33^. Such policies would motivate technological advancements and enforce strict regulations throughout all industries. Developing countries, however, should craft adaptive policy frameworks to keep pace with their rapid growth. This includes multi-sectoral strategies tailored to their unique economic and environmental circumstances, with a priority on acquiring green infrastructure and technology from more advanced nations to accelerate their journey towards sustainable solutions (Figure SI-25).

The technological landscape for environmental solutions among G20 countries also varies greatly^34^. Innovators like Japan and Germany should continue to support research and development, enhancing cooperation across government, academia, and industry to drive progress. Countries trailing in technological advancements need to promote technology growth through specific policies, such as tax incentives for green technology and assistance for startups in sustainability fields. Strategic collaborations with technologically advanced countries are essential to align policy incentives with technology goals, enabling them to contribute meaningfully to the global environmental landscape (Figure SI-27).

Lastly, the trade-to-GDP ratio forecasts for G20 countries demonstrate diverse levels of global market integration^35^. Economies with a significant and growing trade ratio should focus on diversification and establishing robust trade frameworks to lessen the impact of market volatility. Countries with lesser or fluctuating trade ratios should work towards market liberalisation and enhancing trade mechanisms, which includes streamlining trade processes, upgrading logistic networks, and fostering competitive domestic industries for greater participation in global trade. Adaptable policies are paramount to ensure that economies can effectively navigate both domestic and global economic shifts (Figure SI-28).

Time series forecasting for accounting the ecological footprints of G20 nations offers predictive insights into future environmental trends based on historical data, facilitating data-driven decision-making. It serves as an early warning system, aids in efficient resource allocation, and helps track the performance of environmental policies over time. However, its effectiveness is limited by the quality and availability of data, the complexity of ecological systems, and the potential for sudden changes in environmental patterns that traditional models may not capture. Additionally, the process is resource-intensive and may face delays in policy formulation and implementation, reducing its immediacy and relevance. Despite these challenges, this AI-based time series forecasting remains a valuable tool for predicting and managing ecological impacts, provided these limitations are carefully managed.

## Conclusions

This study advances our understanding of forecasting ecological footprint deficits and reserves by focusing on the G20 countries as a representative global sample. It leverages data and methodologies from the Global Footprint Network, employing a univariate approach that relies on existing variables within this framework.

While refining parameters and optimisation could improve the accuracy of this analysis, predicting the future ecological footprint balance (reserve or deficit) of G20 countries remains a complex challenge. Various anticipated factors, such as population growth, economic development, and technological progress will undoubtedly influence ecological footprints. However, unpredictable events, such as the COVID-19 pandemic or the Russia-Ukraine conflict, could intensify resource demands and carbon emissions, further exacerbating ecological footprint deficit.

The forecasting methodologies employed in this work provide a glimpse into the next 30 years, suggesting that countries are likely to continue consuming resources beyond their production capabilities, thereby endangering future generations’ well-being if mitigation actions are not undertaken. This trend appears consistent across both developed and developing G20 nations.

Effectively addressing the ecological footprint in G20 countries requires a comprehensive approach that includes both individual behaviour changes and public policy reforms. Proactive measures by G20 nations, such as investing in renewable energy, improving energy efficiency, and adopting sustainable agricultural practices, could gradually reduce the ecological footprint decline over time.

In summary, the future ecological footprint of G20 countries is expected to be determined by a complex mix of socio-economic, and technological factors, rendering precise predictions difficult. Nevertheless, it is imperative for G20 countries to work collaboratively to tackle these challenges, aiming to minimize resource scarcity and enhance resource resilience. The ecological footprint has global ramifications, and the actions of one nation can significantly affect the environment and natural resources worldwide.

## Methods

### Modelling process

We performed time series forecasting in Python using three different methods: ARIMA, Auto ARIMA, and Prophet. The following steps outline the process we followed:

- Data Collection and Preprocessing: We collected the time series data and loaded it into a pandas DataFrame. We then handled any missing values, outliers, or irregularities in the data using appropriate data preprocessing techniques.
- Data Visualization and Exploratory Data Analysis (EDA): We plotted the time series data to visualize its pattern, seasonality, and trends. Additionally, we conducted exploratory data analysis (EDA) to gain insights into the data and determine if any transformation was needed to achieve stationarity.
- Data Splitting: To evaluate the forecasting models, we split the data into training and test sets. The training set was used to fit the forecasting models, while the test set was used for evaluation.
- For the ARIMA method, we estimated the model order (p, d, q) by analysing ACF and PACF plots. Next, we fitted the ARIMA model to the training data with the selected parameters. We then forecasted future values using the fitted ARIMA model and evaluated the forecast performance on the test set using appropriate evaluation metrics.
- For Auto ARIMA, to automatically select the best (p, d, q) parameters for the ARIMA model, we utilized the pmdarima.auto, arima function based on AIC or BIC criteria. Subsequently, we fitted the Auto ARIMA model to the training data, forecasted future values using the fitted model, and evaluated the forecast performance on the test set.
- For the Prophet method, we created a Prophet model object and prepared the data in the specific format required by Prophet (a DataFrame with "ds" column for dates and "y" column for the target variable). We then fitted the Prophet model to the training data, forecasted future values using the fitted model, and evaluated the forecast performance on the test set.
- Forecast Combination: To combine the forecasts obtained from the three methods (ARIMA, Auto ARIMA, and Prophet), we calculated their average. Additionally, we had the option to weigh the forecasts based on the historical performance of each method.
- Evaluation and Comparison: We compared the performance of each individual method (ARIMA, Auto ARIMA, and Prophet) with the combined forecast using appropriate evaluation metrics such as Mean Squared Error (MSE), Mean Absolute Error (MAE), or Root Mean Squared Error (RMSE).
- Visualization of Results: We visualized the results by plotting the original time series data, the individual forecasts from each method, and the combined forecast, allowing for a visual comparison of their predictions.

The above-described process provided us with a comprehensive and systematic approach to time series forecasting, allowing for evaluation and comparison of the individual methods and the combined forecast.

### Ecological footprint summary

In this analysis we used the original ecological footprint approach, where Biocapacity: is considered as the productive biological area in both land and sea, which is a basic unit in the calculation of sustainability and the ecological footprint of any given area, country or region^36^, this unit area also has the potential capacity to soak up carbon dioxide waste if appropriate management practices are in action. On the other hand, the EF consumption considers the area required by human consumption, which includes the use of natural resources for local production and of course, the difference of those natural resources embedded in import and export trading activities. Hence, if the ecological footprint or consumption of a country is bigger than its biocapacity a negative balance or deficit occur, on the other hand if the consumption is smaller than the biocapacity a reserve or positive balance is accounted. Ecological footprint is measured in standard units called global hectares and the methodology procedure is expressed with the following formula^17^;

$$EF_{C} = EF_{P} + EF_{I} - EF_{E}$$

where $EF_{P}$ the ecological footprint of consumption, $EF_{P}$ the ecological footprint of production, $EF_{I}$ the ecological footprint of imports, $EF_{E}$ the ecological footprint of exports.

### Data

Historical data from calculations by the global ecological networks among the G20 countries have been used for this analysis (Fig SI 1). In the original dataset there are results from the National Footprint Accounts 2019, which includes 196 countries and total "World" for data years 1961 through 2018, the most recent year with complete data (*). From the G20, 18 countries have all records since 1961 to 2018, while records from Russia are also complete, data from 1961 to 1991 corresponds to the USSR period, Saudi Arabia provided records from 1980 ahead. For each nation and year, it is plotted the difference between its Biocapacity (blue) and Ecological Footprint of Consumption (red) as deficit/balance (purple). in these three elements for comparison across different world regions, units of measure are total global hectares per capita (gha/cap), which is a standardised unit by biological productivity across land type.

To update and complete data from the original database, we also incorporated additional indicators from the world bank database. Socio-Economic related: Population (SP.POP.TOTL), Global domestic product (NY.GDP.MKTP.CD), Global domestic product per capita (NY.GDP.PCAP.KD). Energy: kwh per capita (EG.USE.ELEC.KH.PC), kg of oil equivalent per capita (EG.USE.PCAP.KG.OE). Climate change: CO2 emissions in kilotons (EN.ATM.CO2E.KT), CO2 emissions in Megatons per capita (EN.ATM.CO2E.PC). Land area: forest area in km^2^ (AG.LND.FRST.K2), crops area in km^2^()^37^.

Additionally, to create the recommendations as explained in the discussion section, we integrated various indices and data sources to assess the impact of environmental policies and climate change mitigation efforts globally. We utilized the Environmental Policy Stringency Index (EPS) from the OECD, which measures the rigor of environmental regulations across countries by assessing the costs imposed on pollution or environmentally harmful behaviours. This index is invaluable for understanding the differences in policy effectiveness internationally. Furthermore, we examined cross-sectorial policies for climate change mitigation among the G20 countries, as developed by the International Programme for Action on Climate. This analysis, scoring from 1 to 10, reviews over 130 policy variables across 56 major climate actions, offering a comprehensive view of how these nations strive to lower emissions and promote environmental responsibility^31–33^.

Furthermore, we considered patent statistics and indicators to gauge advancements in green technologies and their contribution to environmental policy and innovation. Lastly, the relationship between international trade and GDP for each country was analysed to understand economic impacts tied to environmental actions. These combined efforts aim to provide a holistic understanding of global environmental policy effectiveness and technological innovation in addressing climate change, serving as a critical resource for policymakers, researchers, and advocates in the field^34,35^.

### Time series models

The Autoregressive integrated moving average (ARIMA) was used in choosing a reliable model for this work. ARIMA models are capable of predict future values based on past values. ARIMA makes use of lagged moving averages to smooth time series data. They are widely used in technical analysis to forecast future security prices^30^.

An ARIMA model involves estimation of the parameters which account for the trends and autoregressive (AR) and moving averages (MA) processes. The typical ARIMA (p,d,q) comprises three types of parameters: the AR parameters (p), the number of differencing induced (d), and the MA parameters (q)^38^. When dealing with seasonal time series, seasonal parameters must be incorporated into the model. In general, the order of a SARIMA model is given by (p,d,q)(P,D,Q)s where P, D, and Q represent the seasonal AR order, seasonal integration order, and seasonal MA order, respectively, and where s denotes the period of the season (in the monthly case, s = 12).

Auto ARIMA (Auto-regressive Integrated Moving Average) is a popular forecasting model used in time series analysis. It is an extension of the ARIMA model that automatically selects the best parameters (p, d, q) for the ARIMA model by performing a grid search and finding the combination that minimizes a specified metric (such as AIC or BIC) for the given time series data^39^. The selection of appropriate values for (p, d, q) is crucial for the ARIMA model's performance. This is where Auto ARIMA comes into play. Auto ARIMA performs a grid search over different combinations of (p, d, q) and selects the combination that minimizes the chosen information criterion (e.g., AIC or BIC)^40^. The information criterion takes into account both the model's goodness of fit and its complexity to find the best trade-off between accuracy and parsimony. Auto ARIMA is a useful tool for automating the process of ARIMA model selection, especially when dealing with large datasets or when you are not familiar with the underlying time series patterns. It is widely used in various fields, including finance, economics, sales forecasting, and other areas where time series analysis is crucial for decision-making and predictive modelling.

Along with traditional ARIMA and given the potential scalability of the current information^41^, we also included the Prophet algorithm, which is a modular and simplify procedure based on regression models that allows us to identify and modify parameters for the analysis of large-scale databases^29^. Basically, this forecast evaluation system makes use of simulated predictions to estimate out-off sample performance, which better reflects the available information to the analyst^28^. This time series forecasting method is developed by Facebook's Core Data Science team. It is designed to handle time series data with strong seasonal patterns and multiple sources of uncertainty. Prophet is particularly well-suited for datasets with irregularities, missing values, and outliers, as it employs robust methods to address these issues. Prophet is implemented in Python and has gained popularity in various industries and research fields due to its ease of use, automatic handling of seasonality, and ability to provide uncertainty estimates. It is an excellent choice for medium to long-term forecasting tasks, especially when dealing with time series data with complex seasonal patterns and multiple sources of uncertainty.

For the presentation of our results, we combined (average) the outputs of ARIMA, Auto ARIMA, and Prophet methods because: Each forecasting method has its strengths and weaknesses, and averaging their results can help mitigate any individual method's biases. By combining the forecasts, you reduce the impact of any systematic errors that might be present in one method. On one hand, different forecasting methods can yield different forecasts due to variations in model assumptions and parameter choices. Averaging can help smooth out these variations, resulting in a more stable and robust forecast^42^. On the other hand, ARIMA, Auto ARIMA, and Prophet have different underlying models and approaches to handle various time series patterns. By combining their forecasts, we can capture complementary information from each method, potentially improving overall forecasting accuracy. Forecasting inherently involves uncertainty, and different models can capture different aspects of this uncertainty. By averaging the results, we get a more comprehensive view of the forecast uncertainty and a more reliable prediction interval.

Combining multiple forecasting methods is a form of ensemble forecasting^43,44^. Ensemble methods often perform better than individual methods because they leverage the wisdom of the crowd, making the forecasts more robust and accurate. Sometimes, a single forecasting method may overfit or underfit the data, leading to suboptimal forecasts. By averaging multiple models, you reduce the risk of overfitting and get a more balanced forecast. Averaging results from different methods can provide a sense of validation and confidence in the forecast^45^. If multiple methods agree on a particular trend or pattern, it increases the reliability of the forecast. However, it's essential to note that while averaging can provide benefits, it's not always a guarantee of improved accuracy. The effectiveness of combining forecasts depends on the quality and diversity of the individual models being averaged. If all methods suffer from similar biases or inaccuracies, averaging may not produce significant improvements.

### Supplementary Information

Supplementary Information.

## Data Availability

The datasets used and/or analyzed during the current study available from the corresponding author on reasonable request.
