# Author Correction: Forecasting the ecological footprint of G20 countries in the next 30 years

**DOI:** 10.1038/s41598-024-63485-y

**Published:** 2024-05-31

**Authors:** Rafael M. Eufrasio Espinosa, S. C. Lenny Koh

**Affiliations:** Advanced Resource Efficiency Centre, Management School, University of Sheffield, Sheffield, UK

Correction to: *Scientific* *Reports* 10.1038/s41598-024-57994-z, published online 09 April 2024

The Funding section in the original version of this Article was omitted. The Funding section now reads:

“The University of Sheffield Institutional Open Access Fund supported the Open Access of this paper.”

The original Article has been corrected.

