# Supplementary material for: Forecasting the ecological footprint of G20 countries in the next 30 years
Source: Sci Rep. 2024 Apr 9;14:8298. doi: 10.1038/s41598-024-57994-z (PMC11004171; doi:10.1038/s41598-024-57994-z)
Supplement: Supplementary file 1 — Supplementary Information. [file 41598_2024_57994_MOESM1_ESM.docx]

**Supplementary Information**

Forecasting the ecological footprint of G20 countries in the next 30 years

Rafael M. Eufrasio^1^ and S.C. Lenny Koh^1^

Advanced Resource Efficiency Centre, Management School, University of Sheffield

[**Figure SI 1** Historical Records G20 EF 3](#_Toc161062054)

[**Figure SI 2** Population ARIMA forecast 4](#_Toc161062055)

[**Figure SI 3** Population Auto-ARIMA forecast 5](#_Toc161062056)

[**Figure SI 4** Population Prophet forecast 6](#_Toc161062057)

[**Figure SI 5** GDP ARIMA forecast 7](#_Toc161062058)

[**Figure SI 6** GDP AUTO-ARIMA forecast 8](#_Toc161062059)

[**Figure SI 7** GDP Prophet forecast 9](#_Toc161062060)

[**Figure SI 8** Emissions ARIMA forecast 10](#_Toc161062061)

[**Figure SI 9** Emissions AUTO-ARIMA forecast 11](#_Toc161062062)

[**Figure SI 10** Emissions Prophet forecast 12](#_Toc161062063)

[**Figure SI 11** EF Consumption ARIMA forecast 13](#_Toc161062064)

[**Figure SI 12** EF Consumption AUTO-ARIMA forecast 14](#_Toc161062065)

[**Figure SI 13** EF Consumption Prophet forecast 15](#_Toc161062066)

[**Figure SI 14** Biocapacity ARIMA forecast 16](#_Toc161062067)

[**Figure SI 15** Biocapacity AUTO-ARIMA forecast 17](#_Toc161062068)

[**Figure SI 16** Biocapacity Prophet forecast 18](#_Toc161062069)

[**Figure SI 17** Forest ARIMA forecast 19](#_Toc161062070)

[**Figure SI 18** Forest AUTO-ARIMA forecast 20](#_Toc161062071)

[**Figure SI 19** Forest Prophet forecast 21](#_Toc161062072)

[**Figure SI 20** Crops ARIMA forecast 22](#_Toc161062073)

[**Figure SI 21** Crops AUTO-ARIMA forecast 23](#_Toc161062074)

[**Figure SI 22** Crops Prophet forecast 24](#_Toc161062075)

[**Figure SI 23** Human Development Index forecast (HDI) 25](#_Toc161062076)

[**Figure SI 24** Environmental Policy Stringency (EPS) index 33](#_Toc161062077)

[**Figure SI 25** Cross Sectorial Environmental Policies for climate change 34](#_Toc161062078)

[**Figure SI 26** International Environmental Policies for climate change 35](#_Toc161062079)

[**Figure SI 27** Development of Technologies for Environmental Management 36](#_Toc161062080)

[**Figure SI 28** International Trade 37](#_Toc161062081)

[**Table SI 1** Population forecast model evaluation (avg) 26](#_Toc161062082)

[**Table SI 2** GDP forecast model evaluation (avg) 26](#_Toc161062083)

[**Table SI 3** Emissions kt model evaluation (avg) 27](#_Toc161062084)

[**Table SI 4** Biocapacity forecast model evaluation (avg) 27](#_Toc161062085)

[**Table SI 5** Forest km2 forecast model evaluation (avg) 28](#_Toc161062086)

[**Table SI 6** Crops km2 forecast model evaluation (avg) 28](#_Toc161062087)

[**Table SI 7** kwh per capita forecast model evaluation (avg) 29](#_Toc161062088)

[**Table SI 8** kg oileq PerCapita mode forecast evaluation (avg) 29](#_Toc161062089)

[**Table SI 9** emissions Mt cap model forecast evaluation (avg) 30](#_Toc161062090)

[**Table SI 10** gdp PerCap model forecast evaluation (avg) 30](#_Toc161062091)

[**Table SI 11** Area PerCap model forecast evaluation (avg) 31](#_Toc161062092)

[**Table SI 11** Biocap PerCap model forecast evaluation (avg) 31](#_Toc161062093)

[**Table SI 13** EFConsPerCap model forecast evaluation (avg) 32](#_Toc161062094)

|  |
| --- |

**Figure SI 1** Historical Records G20 EF

This plot shows the historical footprint balance of the G20 countries, biocapacity per capita, consumption per capita and their corresponding deficit/balance, all units are in global hectares per capita. Source; based on Global Footprint Network

## Population

| 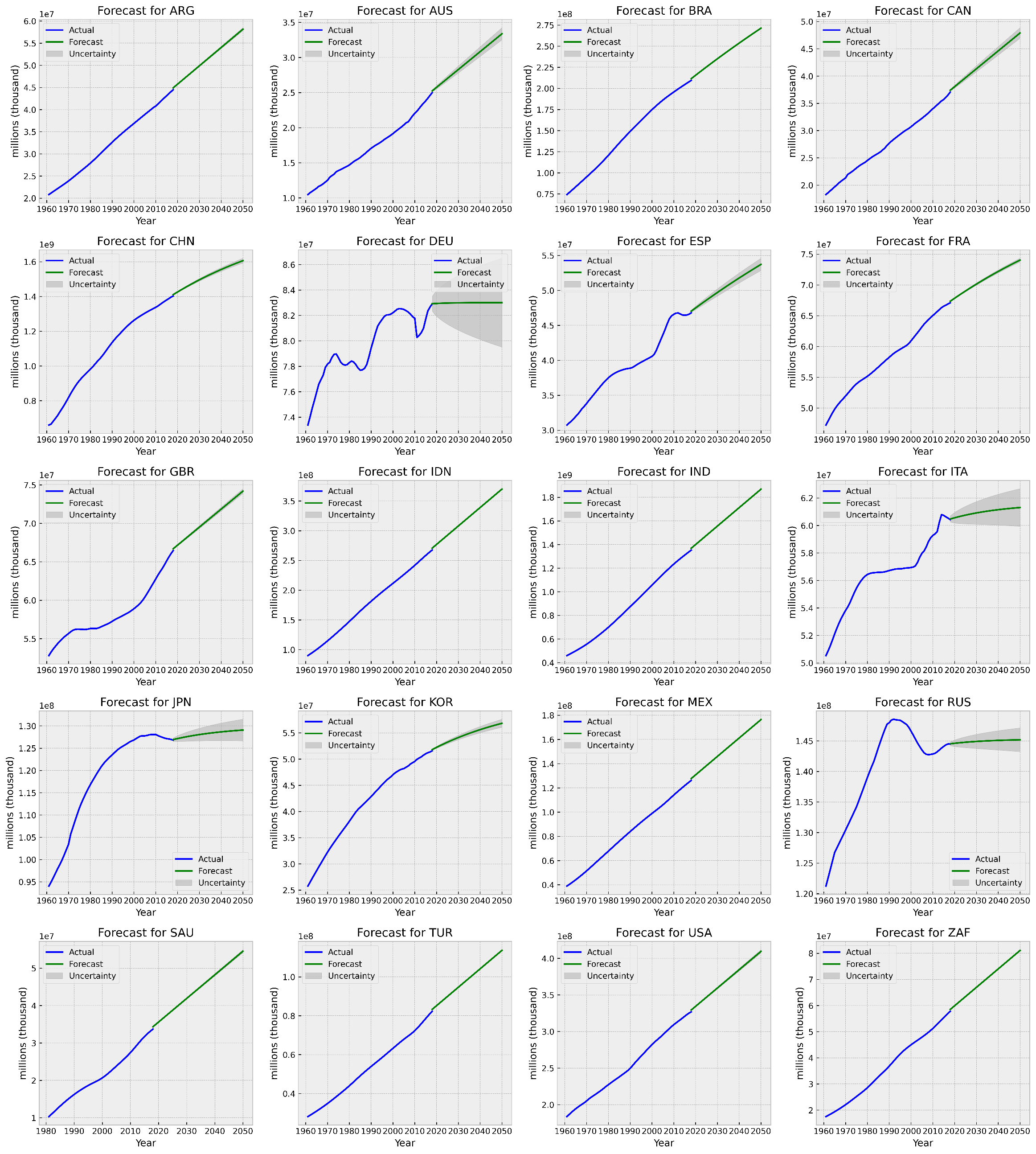 |
| --- |

**Figure SI 2** Population ARIMA forecast

| 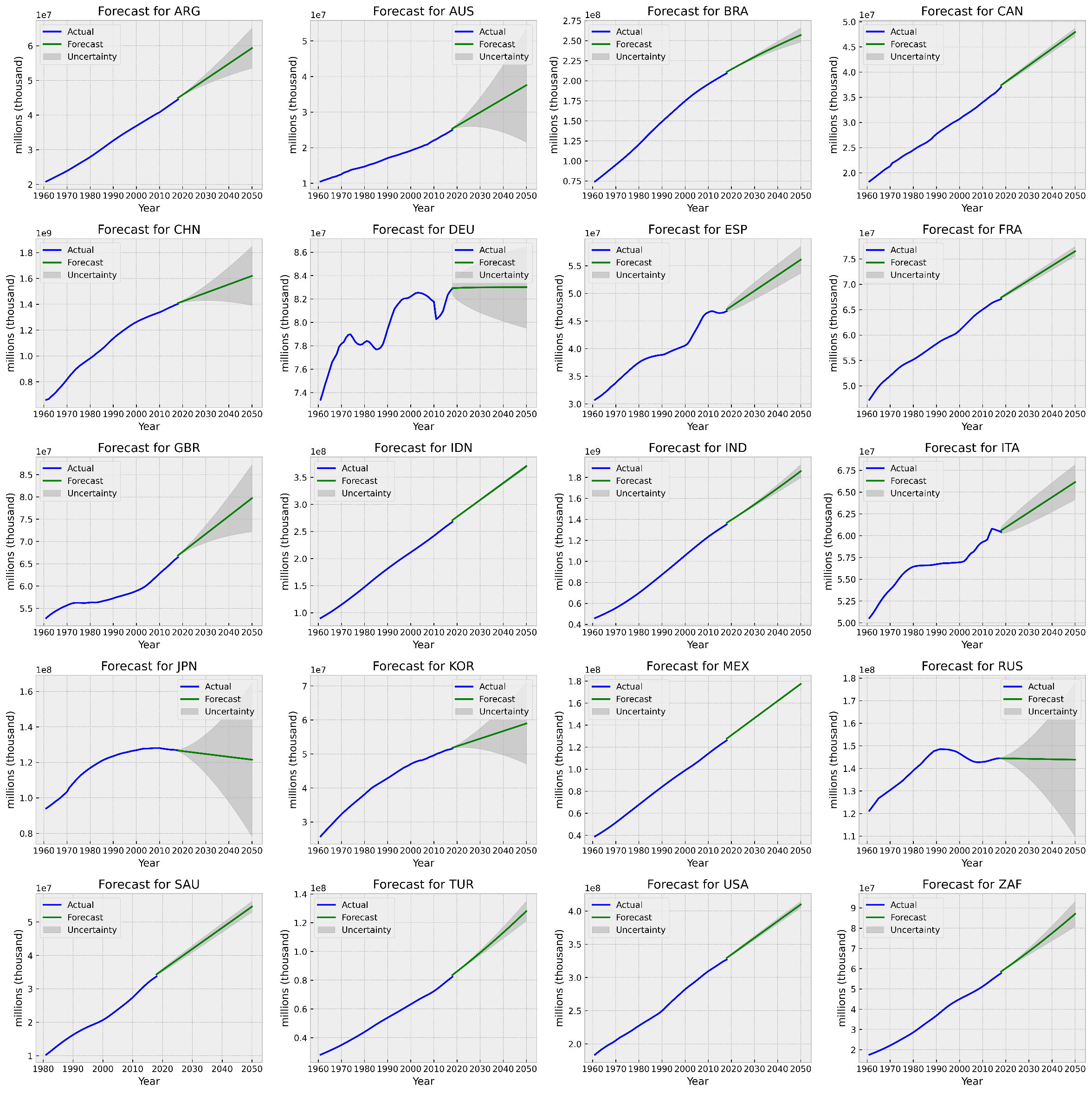 |
| --- |

**Figure SI 3** Population Auto-ARIMA forecast

| 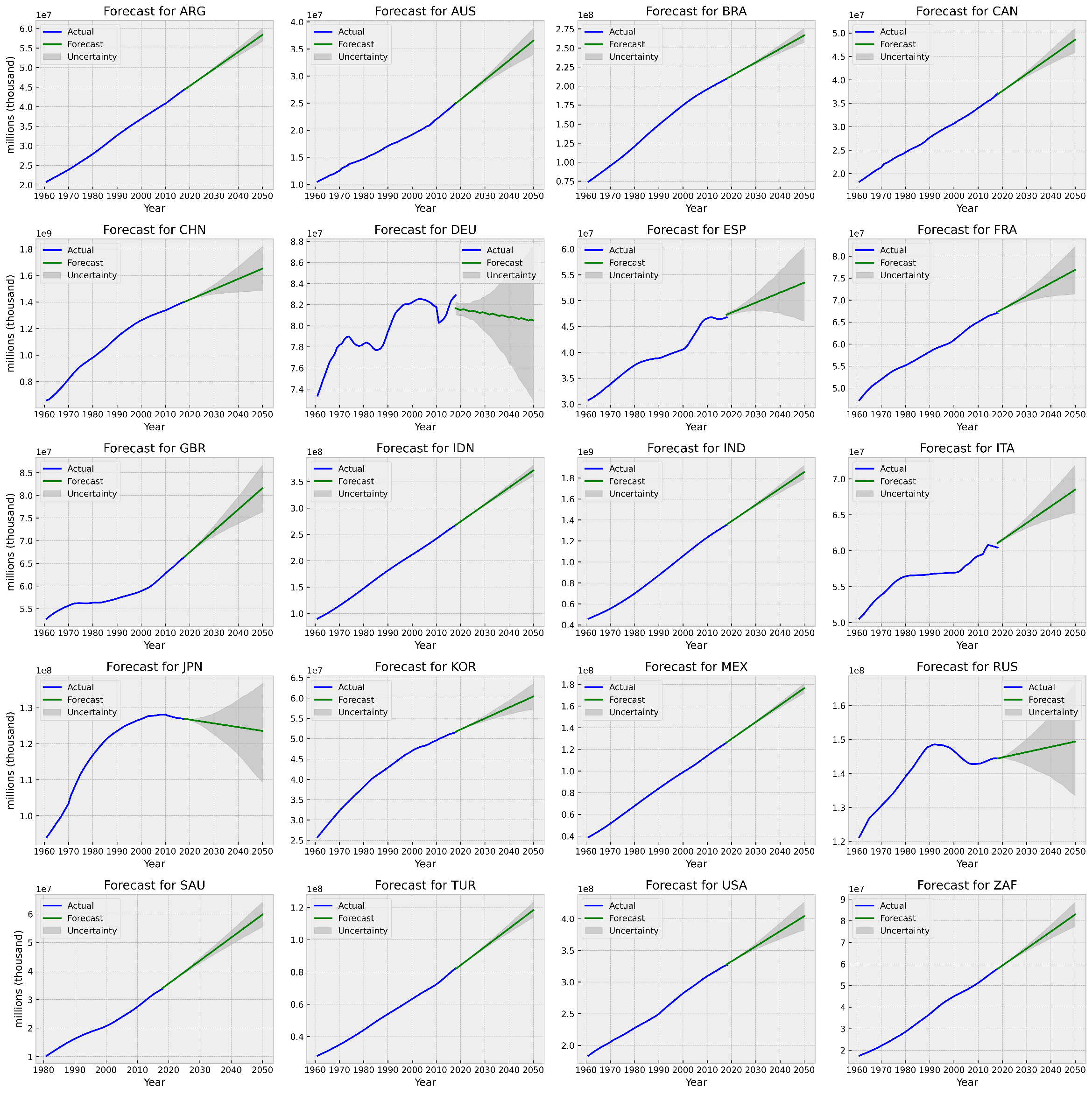 |
| --- |

**Figure SI 4** Population Prophet forecast

## GDP

| 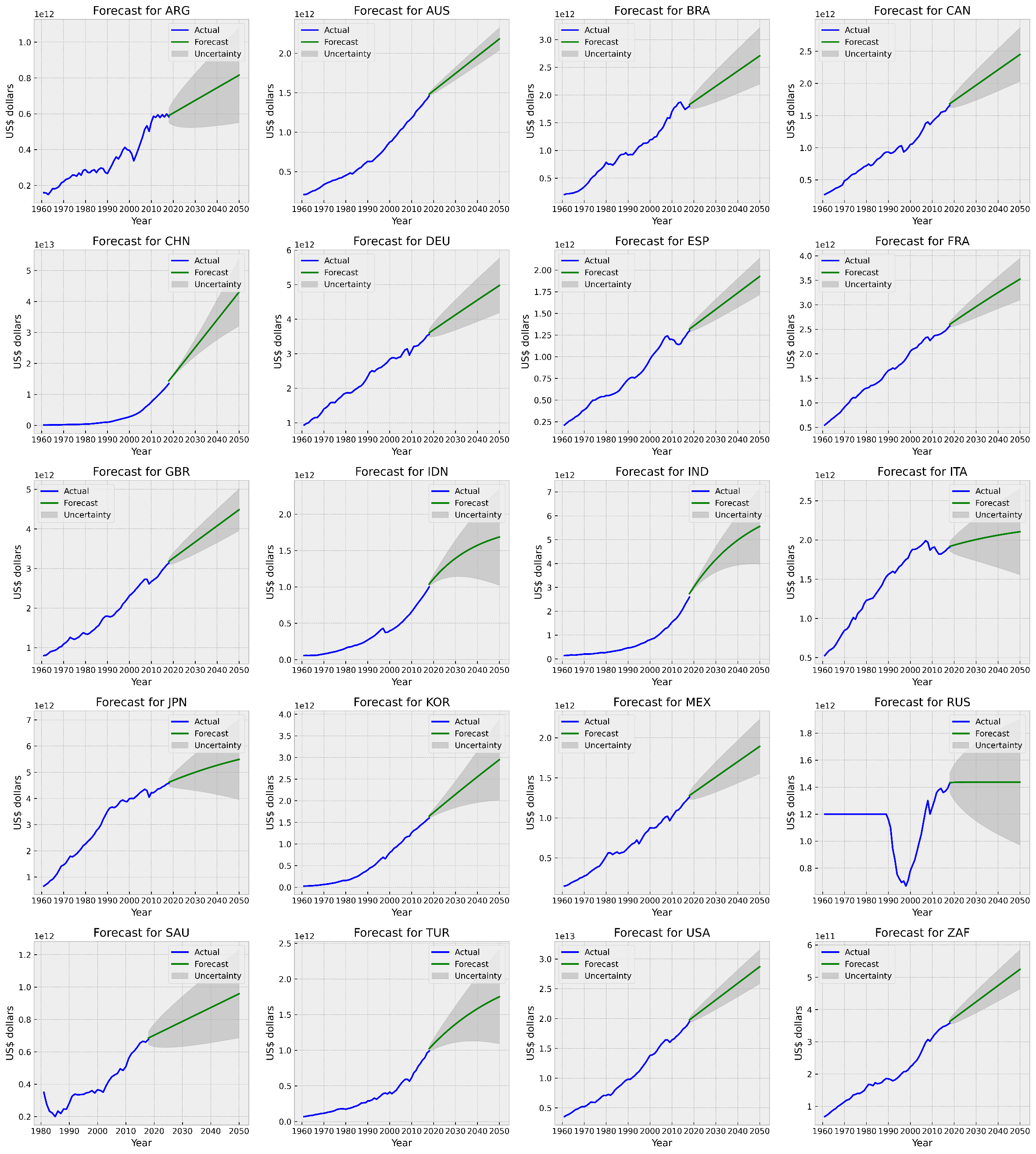 |
| --- |

**Figure SI 5** GDP ARIMA forecast

| 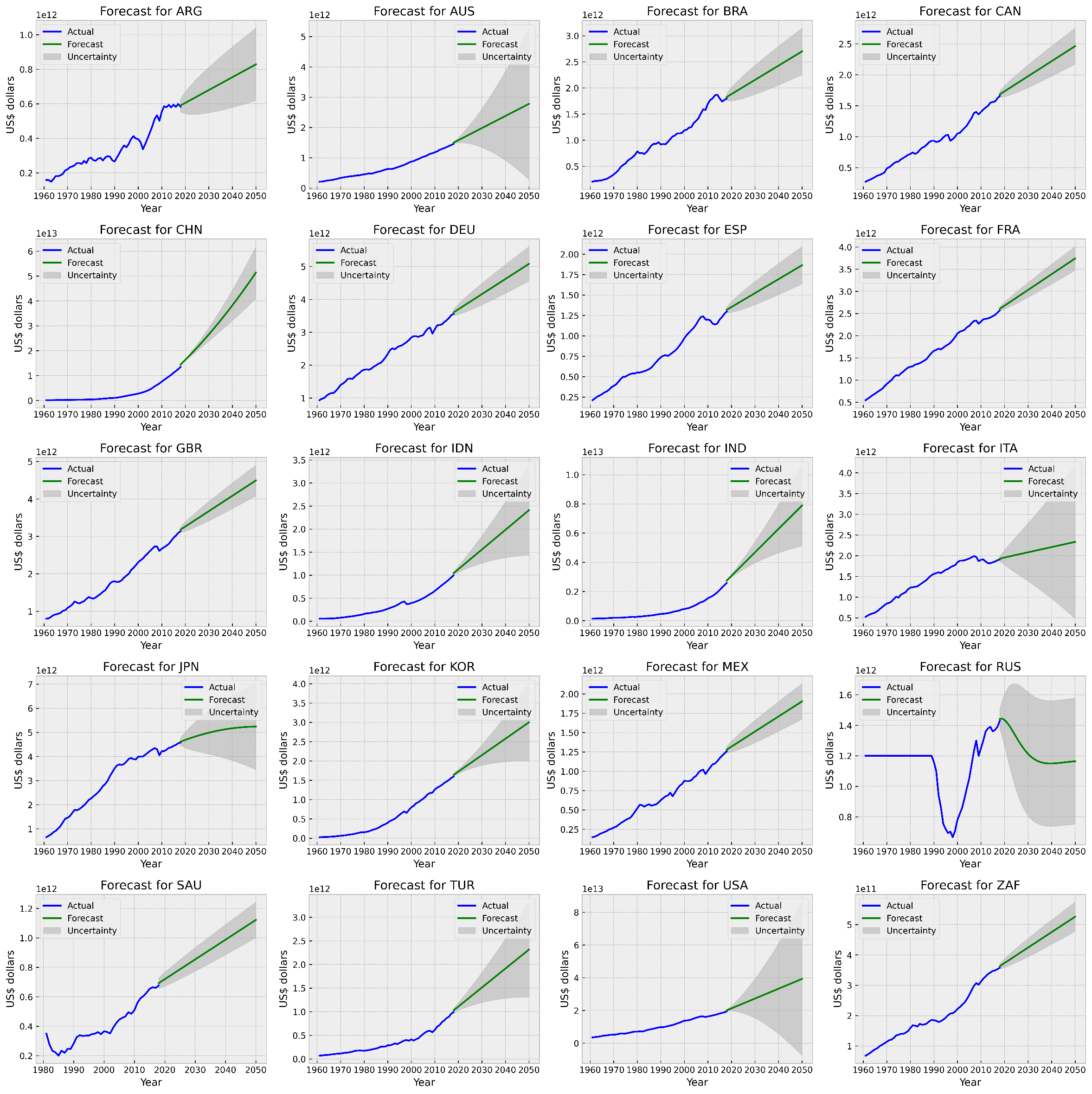 |
| --- |

**Figure SI 6** GDP AUTO-ARIMA forecast

| 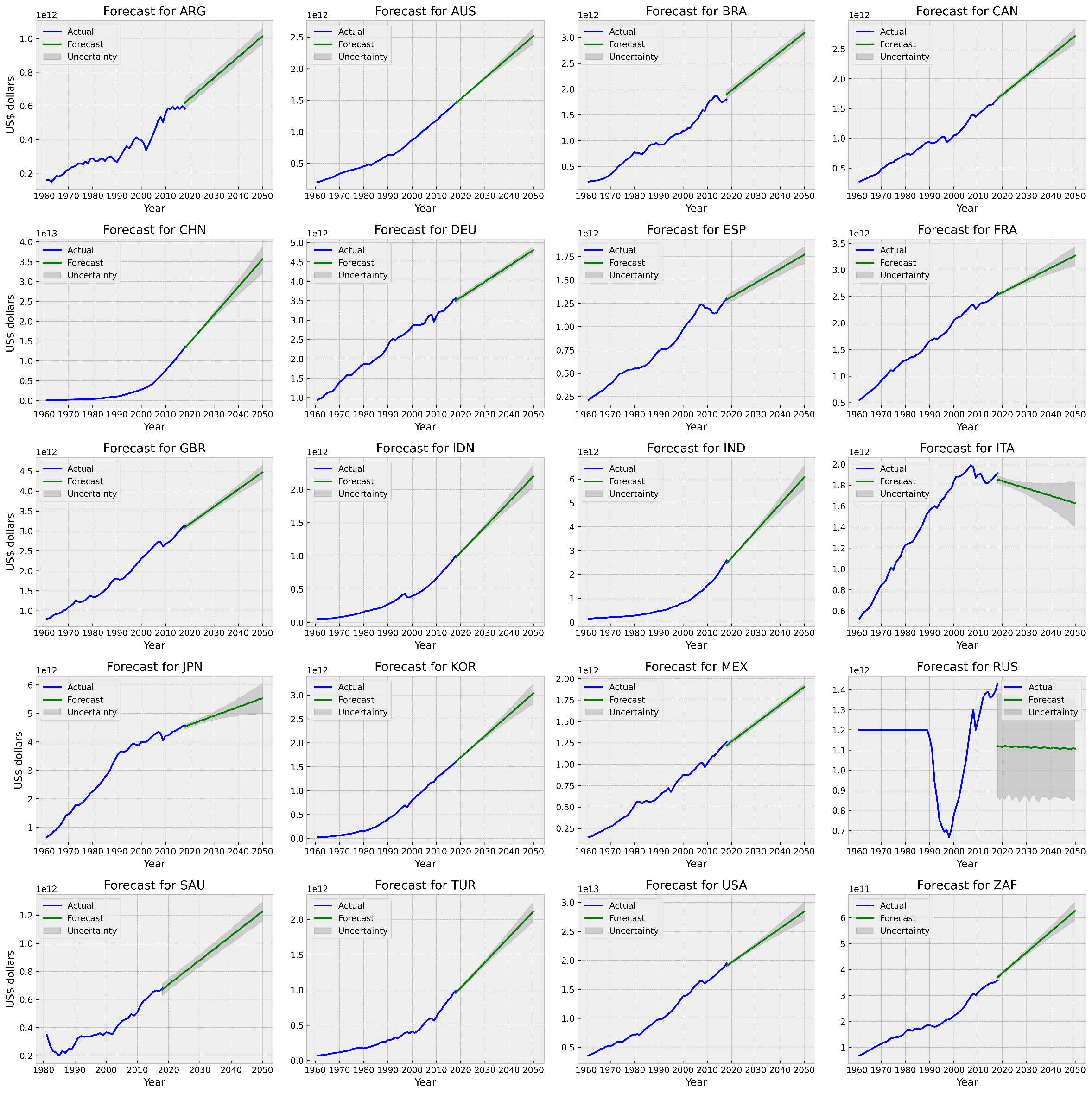 |
| --- |

**Figure SI 7** GDP Prophet forecast

## Emissions

| 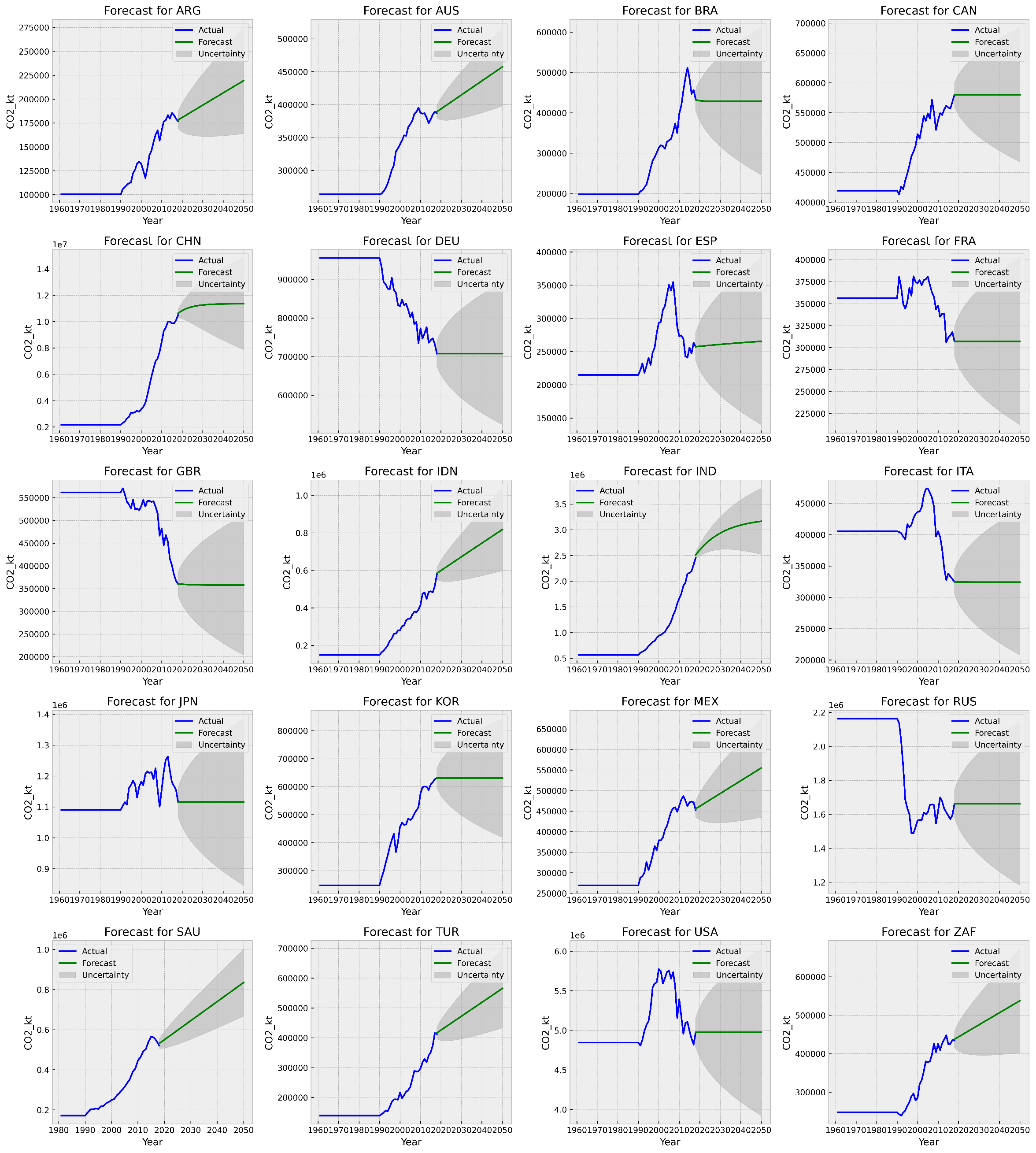 |
| --- |

**Figure SI 8** Emissions ARIMA forecast

| 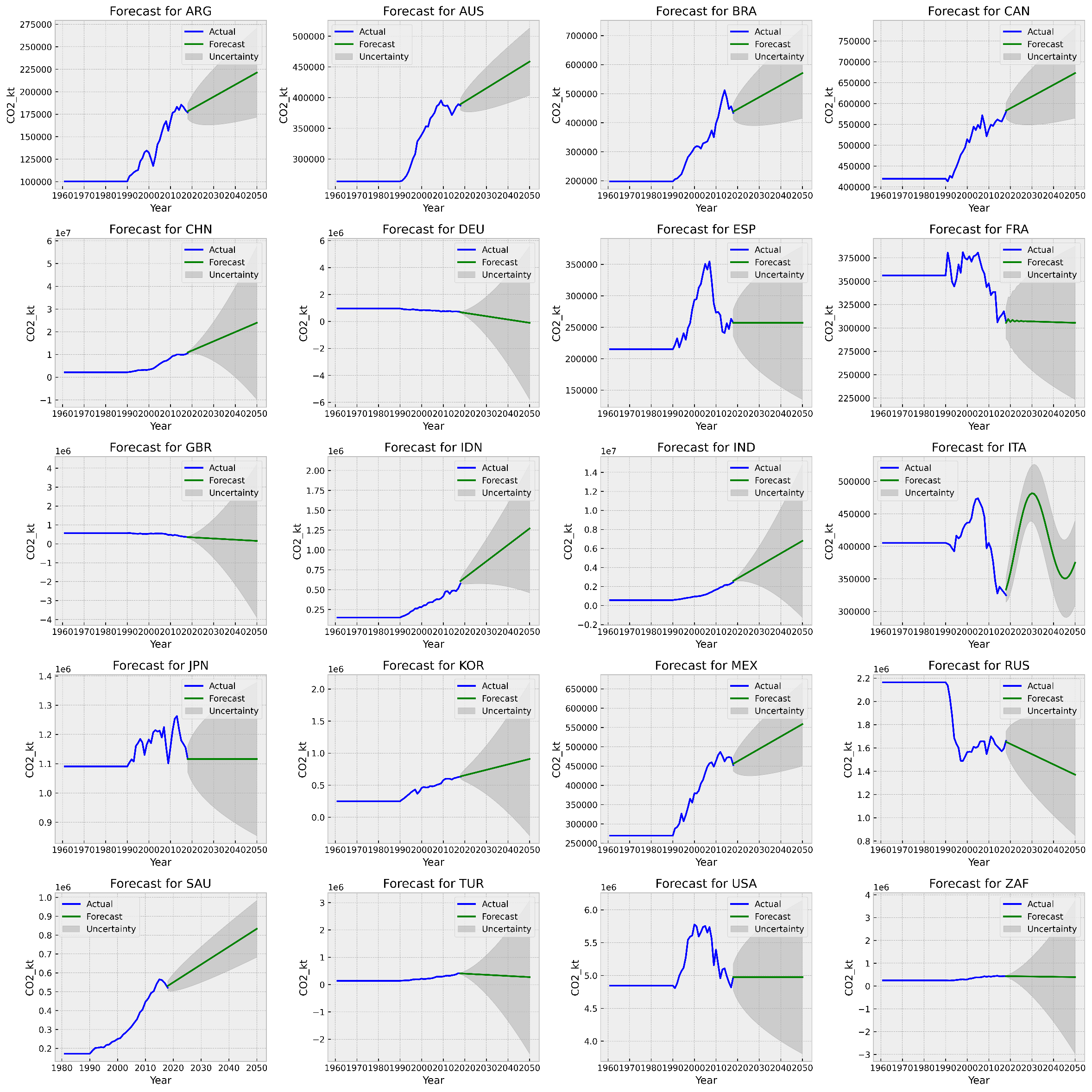 |
| --- |

**Figure SI 9** Emissions AUTO-ARIMA forecast

| 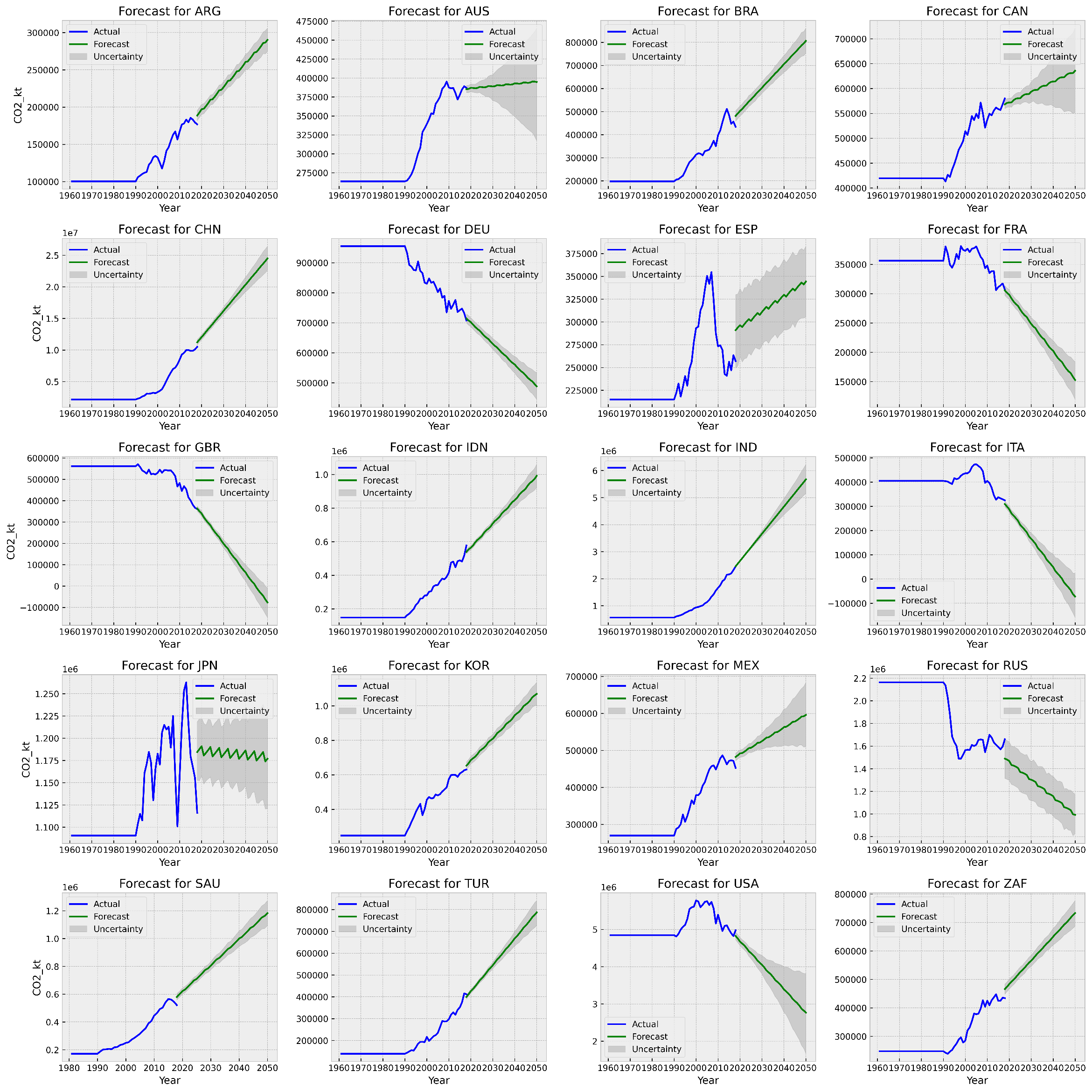 |
| --- |

**Figure SI 10** Emissions Prophet forecast

## Consumption

| 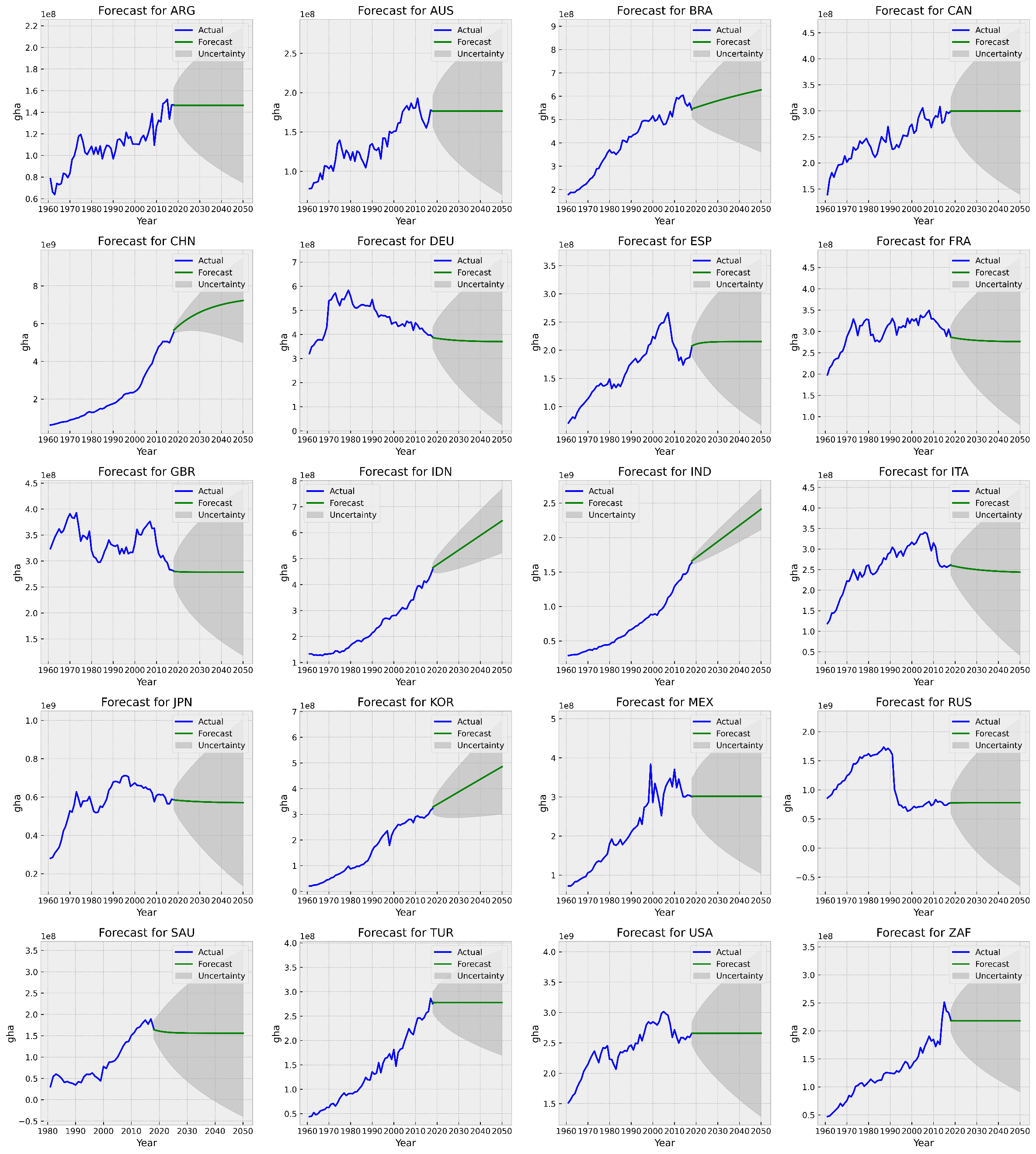 |
| --- |

**Figure SI 11** EF Consumption ARIMA forecast

| 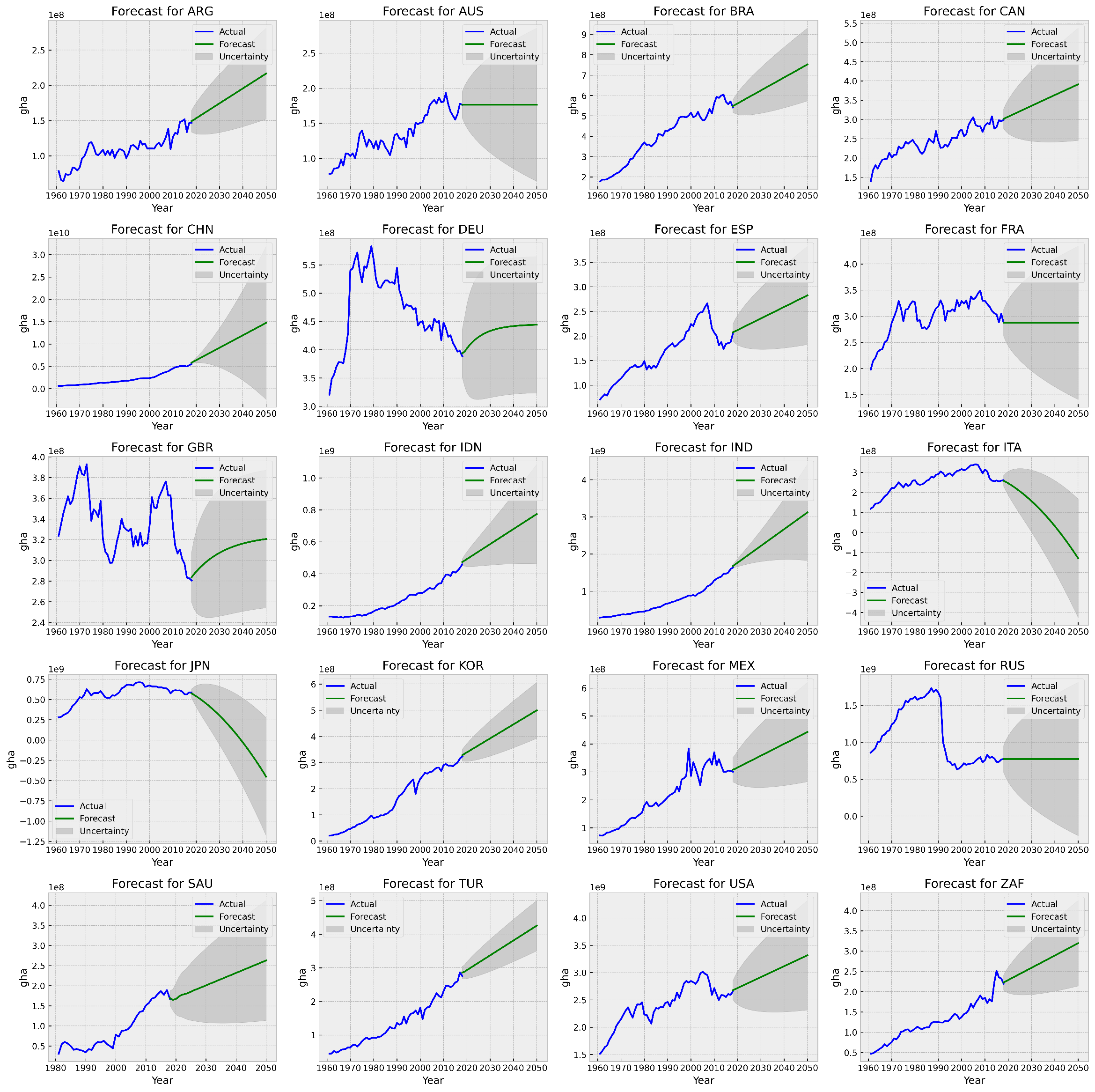 |
| --- |

**Figure SI 12** EF Consumption AUTO-ARIMA forecast

| 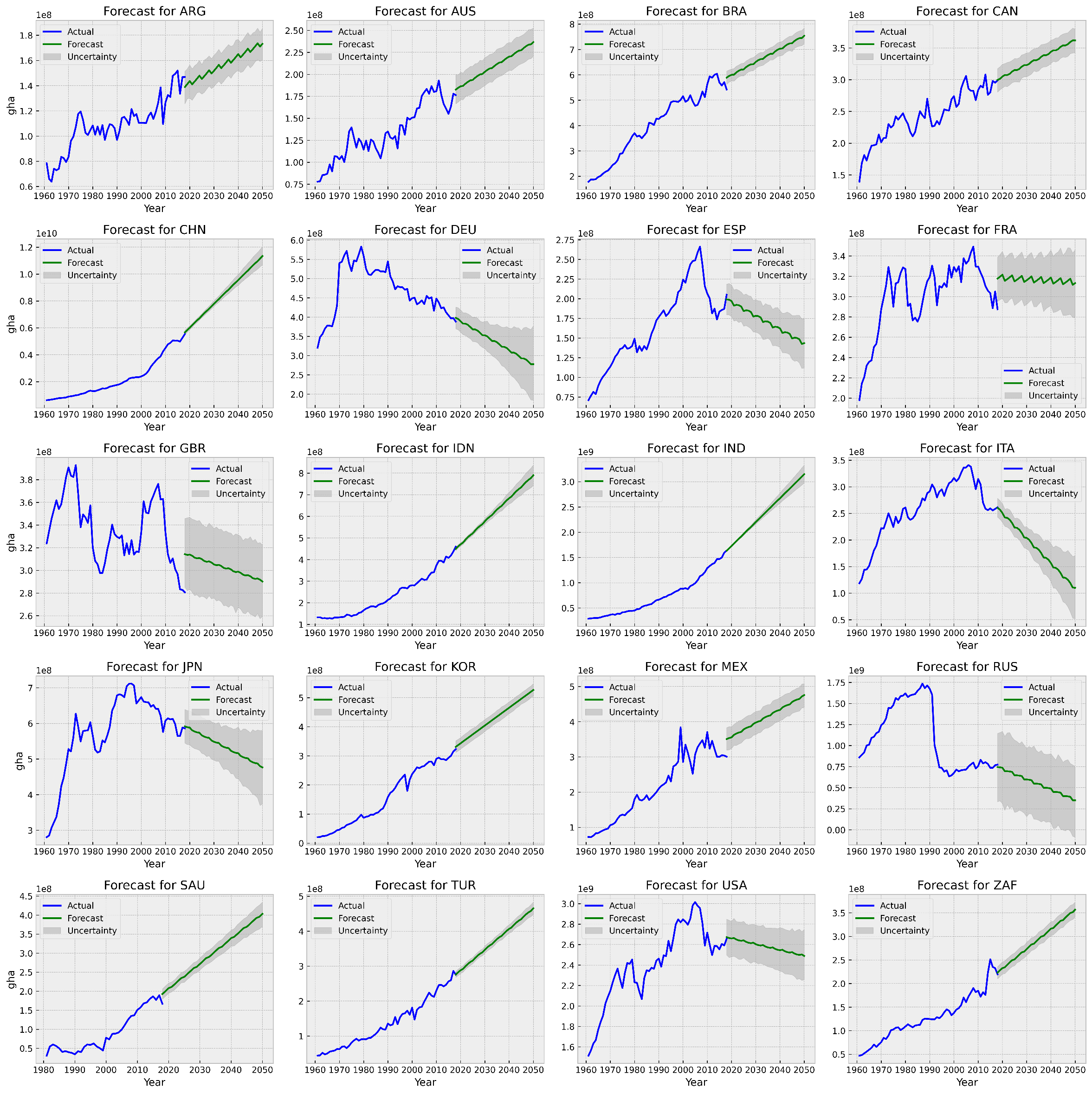 |
| --- |

**Figure SI 13** EF Consumption Prophet forecast

## Biocapacity

| 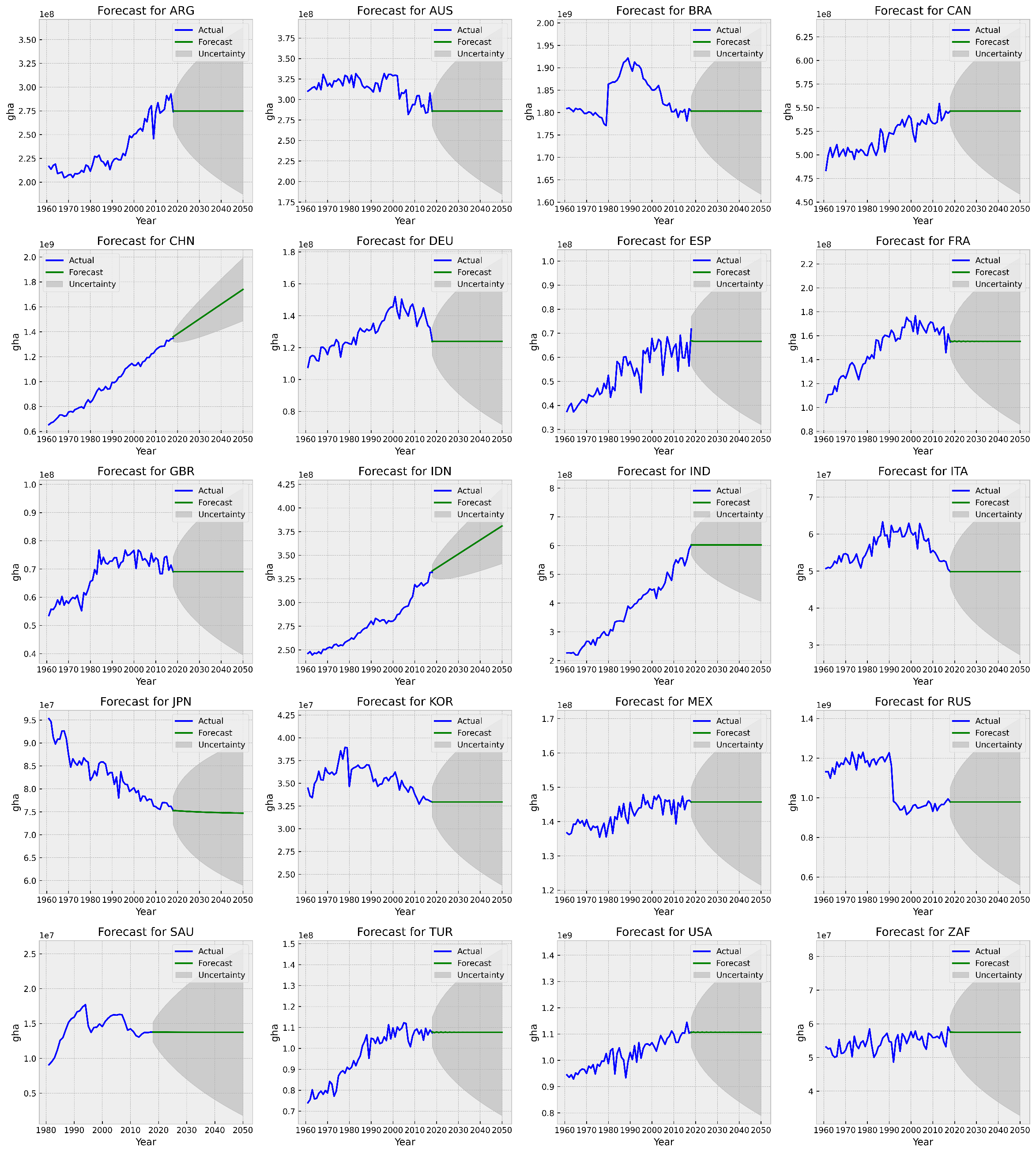 |
| --- |

**Figure SI 14** Biocapacity ARIMA forecast

| 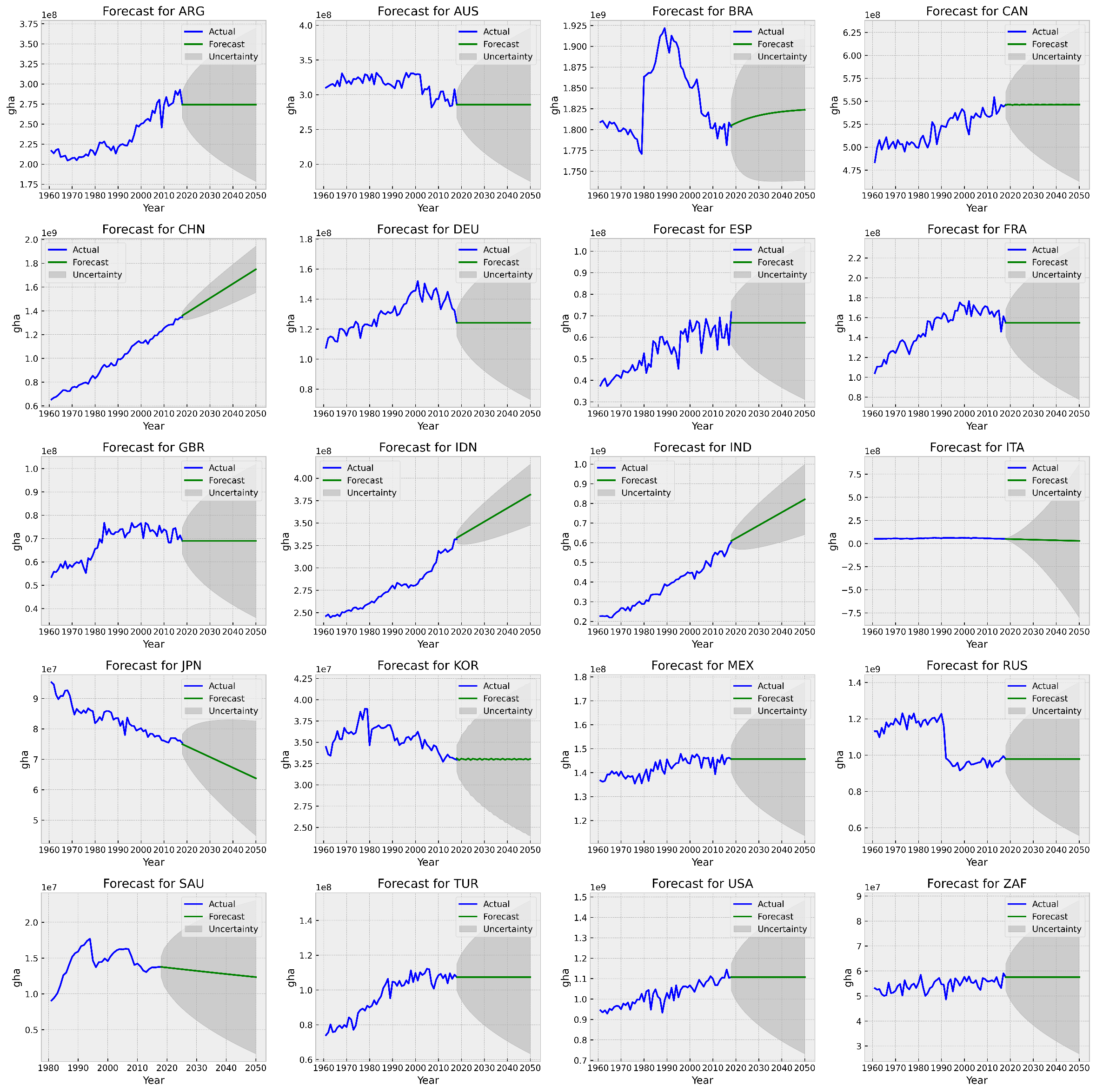 |
| --- |

**Figure SI 15** Biocapacity AUTO-ARIMA forecast

| 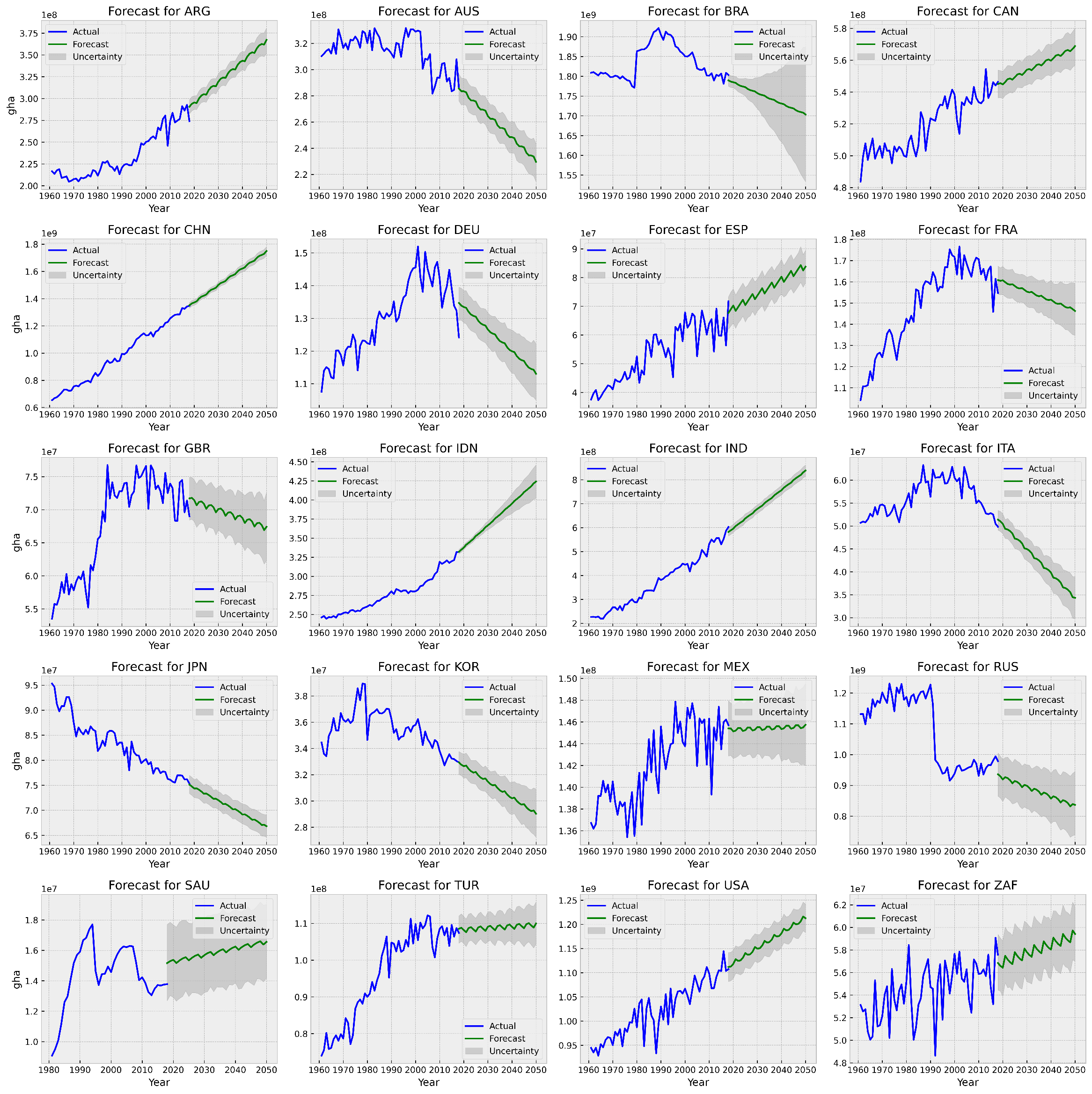 |
| --- |

**Figure SI 16** Biocapacity Prophet forecast

## Forest

| 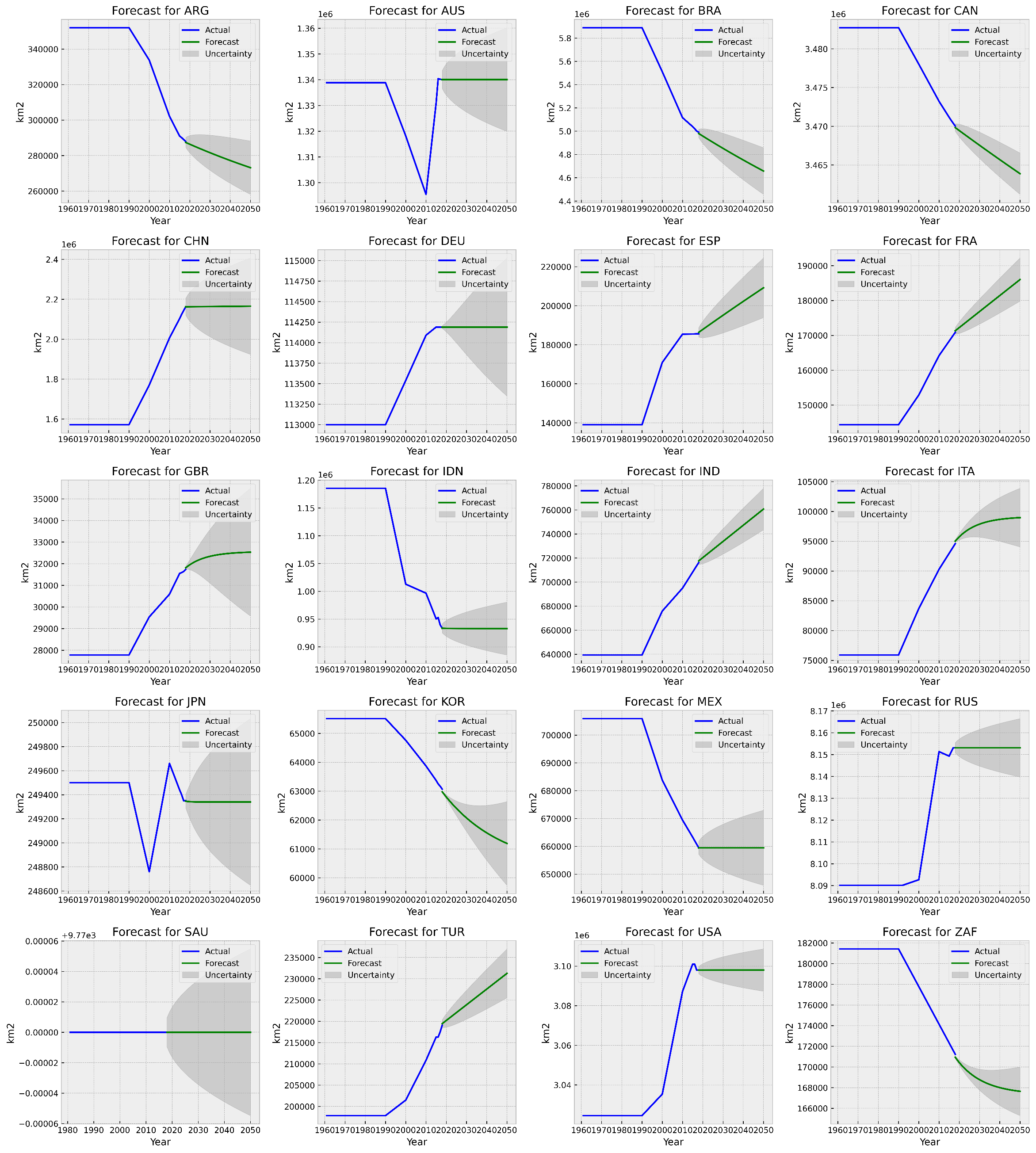 |
| --- |

**Figure SI 17** Forest ARIMA forecast

| 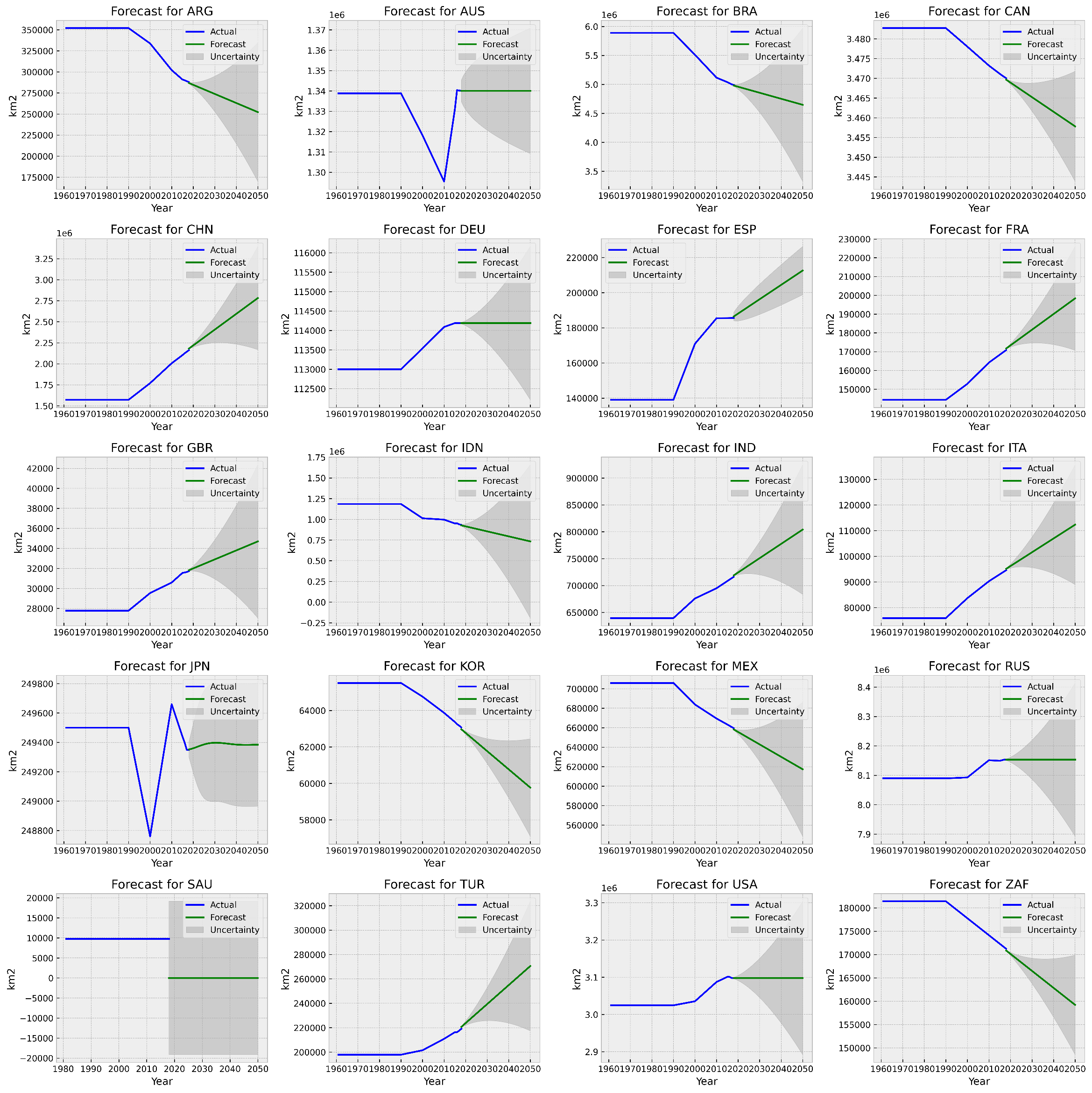 |
| --- |

**Figure SI 18** Forest AUTO-ARIMA forecast

| 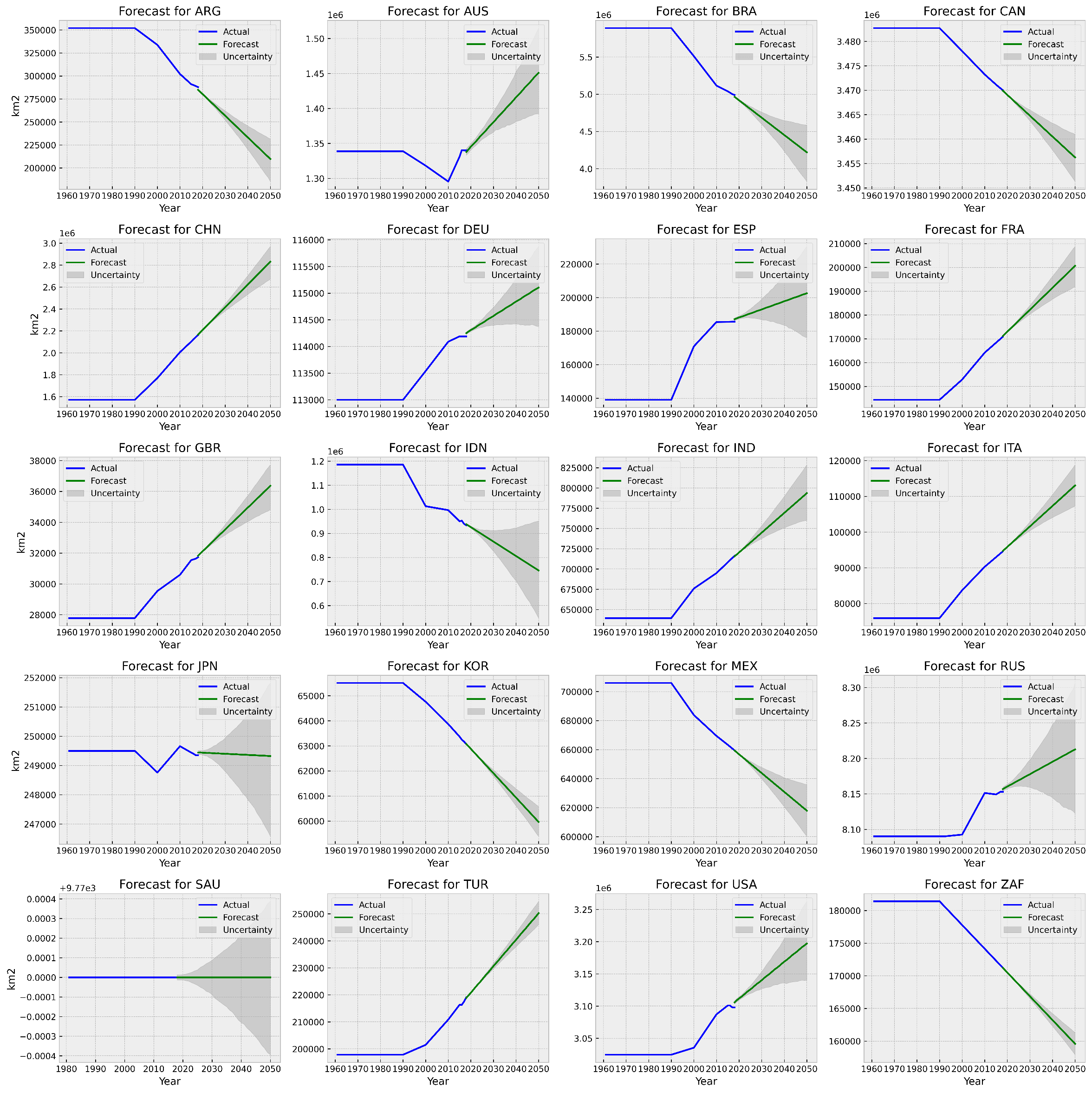 |
| --- |

**Figure SI 19** Forest Prophet forecast

## Crops

| 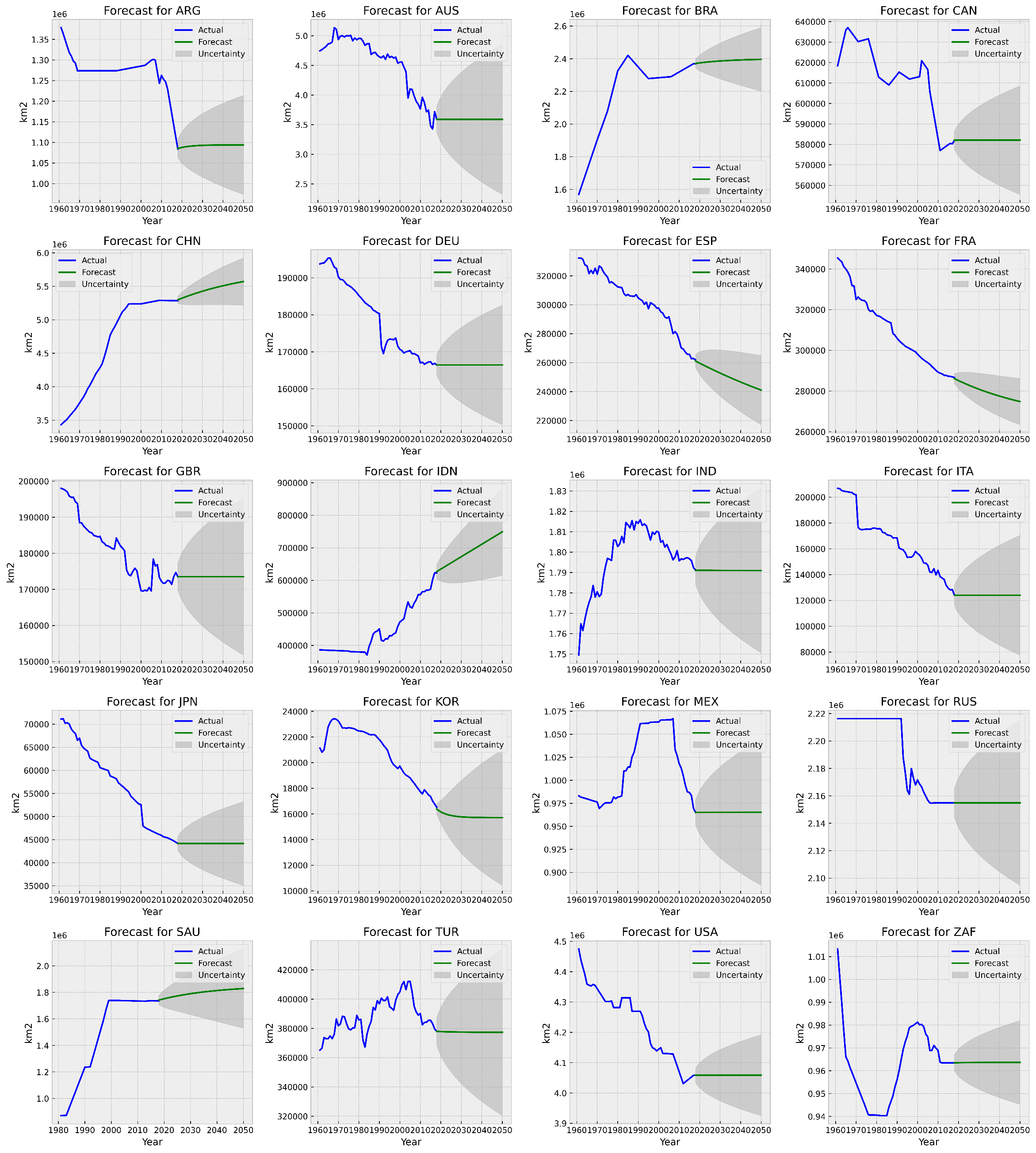 |
| --- |

**Figure SI 20** Crops ARIMA forecast

| 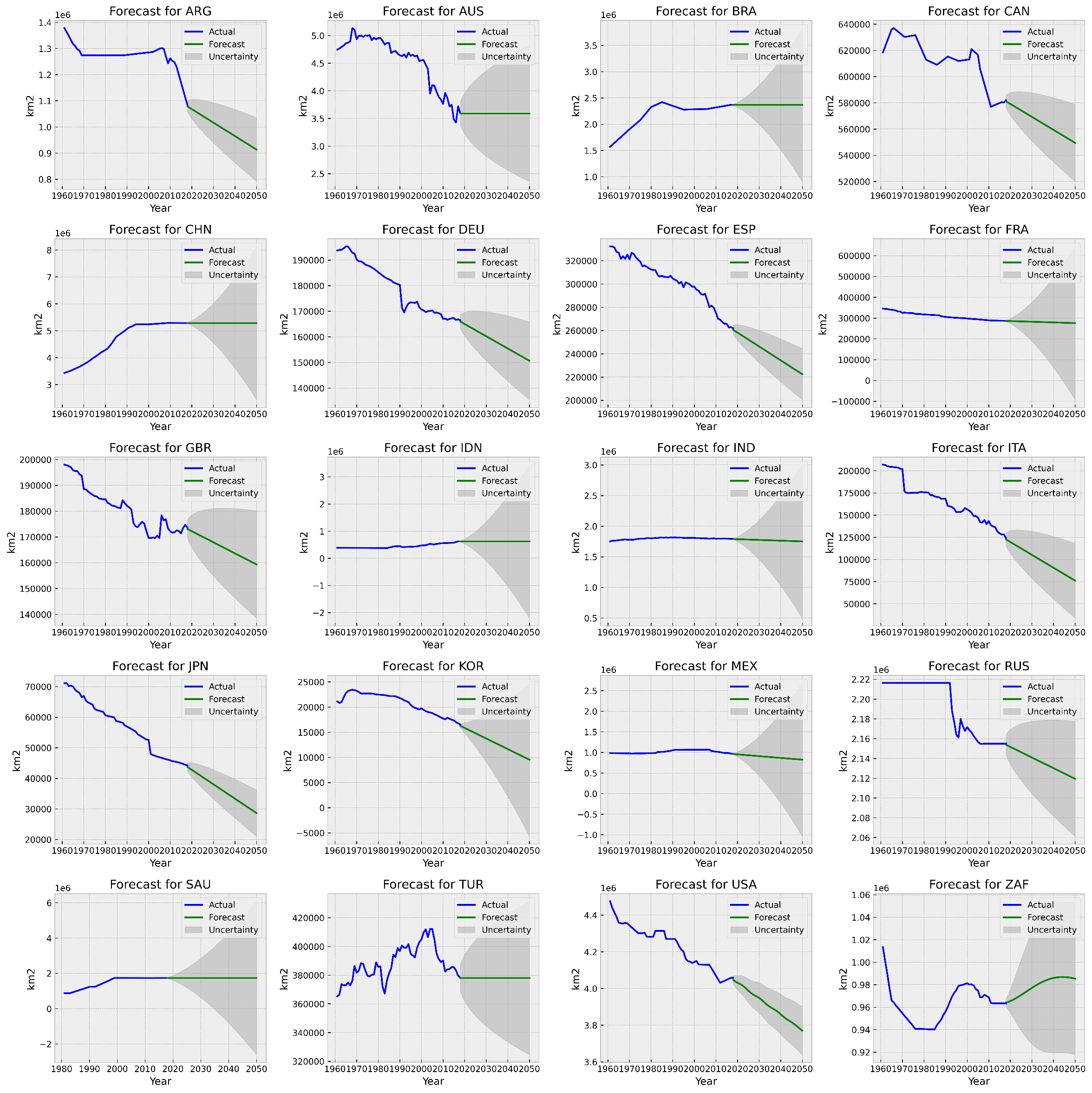 |
| --- |

**Figure SI 21** Crops AUTO-ARIMA forecast

| 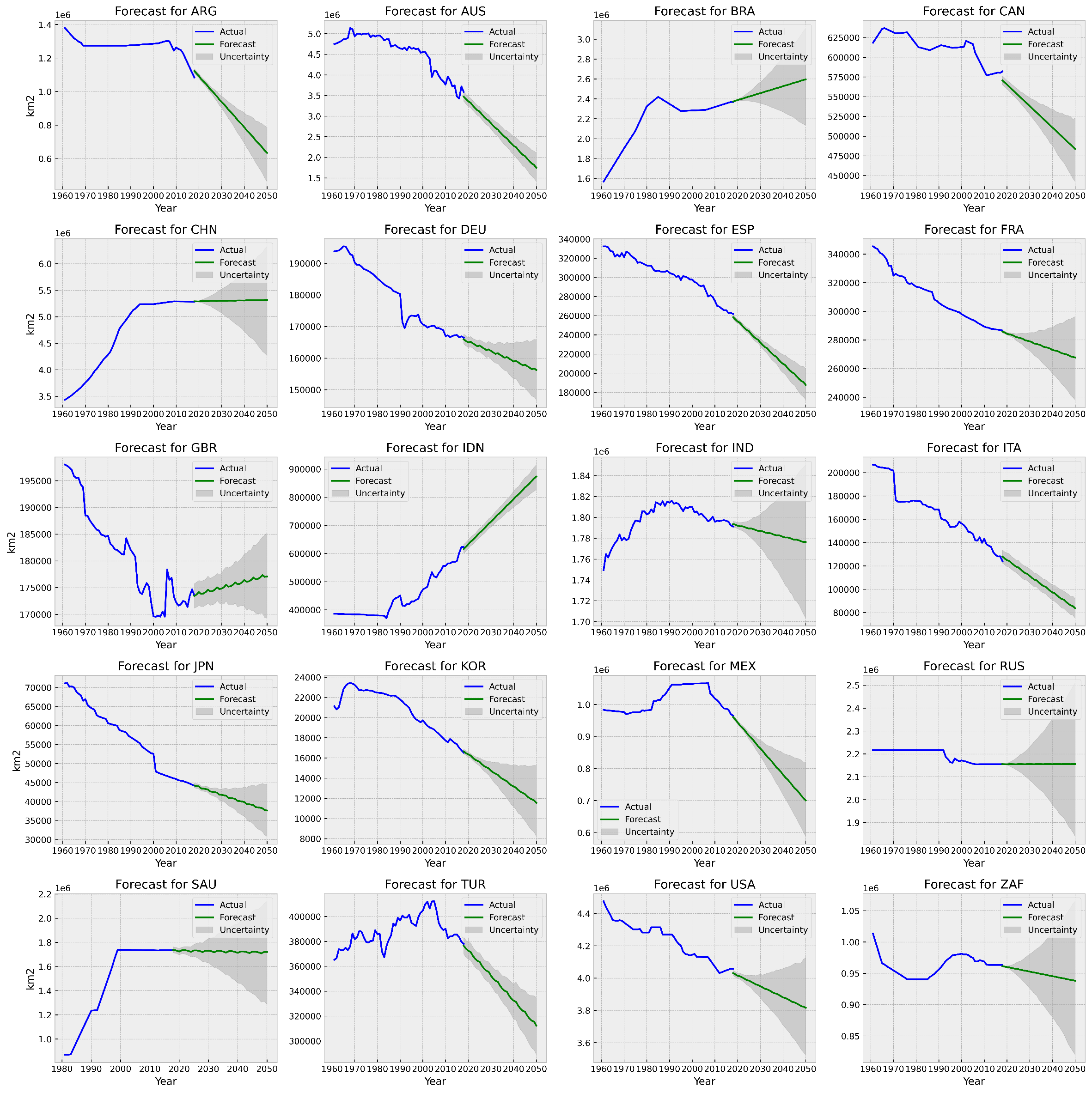 |
| --- |

**Figure SI 22** Crops Prophet forecast

| 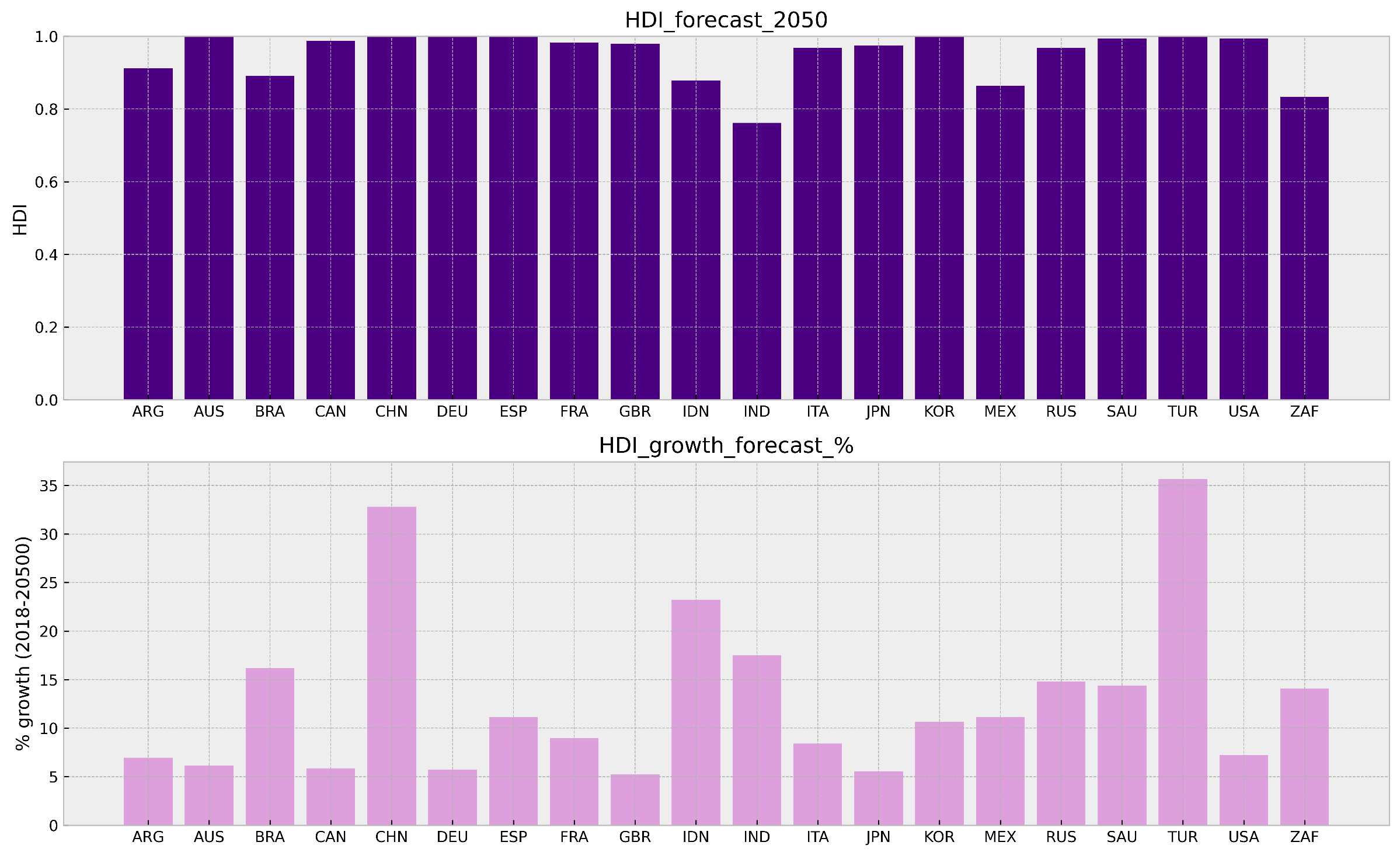 |
| --- |

**Figure SI 23** Human Development Index forecast (HDI)

## Model Accuracy

**Table SI 1** Population forecast model evaluation (avg)

| **Country** | **MAE** | **MSE** | **RMSE** | **MAPE** |
| --- | --- | --- | --- | --- |
| ARG | 97324.43 | 1.69E+10 | 129621.2 | 0.228094 |
| AUS | 766263.8 | 9.2E+11 | 958986.5 | 3.259769 |
| BRA | 5055074 | 3.59E+13 | 5988321 | 2.502804 |
| CAN | 345244.7 | 2.08E+11 | 438744 | 0.9722 |
| CHN | 21229454 | 7.16E+14 | 23263814 | 1.558945 |
| DEU | 1639326 | 4.25E+12 | 1947192 | 2.014287 |
| ESP | 1991704 | 4.62E+12 | 2125482 | 4.322238 |
| FRA | 871777.7 | 1.06E+12 | 984851 | 1.326137 |
| GBR | 2565836 | 8.96E+12 | 2955786 | 3.98809 |
| IDN | 875696.4 | 1.68E+12 | 1093605 | 0.343983 |
| IND | 14845307 | 3.99E+14 | 18855948 | 1.149645 |
| ITA | 1412814 | 3.26E+12 | 1574434 | 2.358035 |
| JPN | 2489616 | 8.79E+12 | 2957912 | 1.955083 |
| KOR | 713660.1 | 7.38E+11 | 801389.3 | 1.417698 |
| MEX | 511407.9 | 4.68E+11 | 596426.6 | 0.429988 |
| RUS | 3484519 | 2.62E+13 | 4334657 | 2.422964 |
| SAU | 1420783 | 3.12E+12 | 1610865 | 4.516905 |
| TUR | 638075.3 | 8.81E+11 | 903968.3 | 0.817822 |
| USA | 2029858 | 9.26E+12 | 2374536 | 0.644429 |
| ZAF | 570777.8 | 6.84E+11 | 705645.5 | 1.061444 |

**Table SI 2** GDP forecast model evaluation (avg)

| **Country** | **MAE** | **MSE** | **RMSE** | **MAPE** |
| --- | --- | --- | --- | --- |
| ARG | 1.37E+11 | 2.2E+22 | 1.46E+11 | 24.55575 |
| AUS | 6.21E+10 | 7.53E+21 | 7.03E+10 | 4.8373 |
| BRA | 2.15E+11 | 5.59E+22 | 2.36E+11 | 12.54724 |
| CAN | 1.75E+11 | 3.61E+22 | 1.85E+11 | 11.83764 |
| CHN | 2.44E+12 | 9.08E+24 | 2.93E+12 | 25.33121 |
| DEU | 8.63E+10 | 1.25E+22 | 9.98E+10 | 2.715993 |
| ESP | 8.69E+10 | 1.15E+22 | 1.03E+11 | 7.250385 |
| FRA | 9.65E+10 | 1.37E+22 | 1.13E+11 | 3.996181 |
| GBR | 9.11E+10 | 1.33E+22 | 1.03E+11 | 3.256057 |
| IDN | 1.78E+11 | 4.62E+22 | 2.14E+11 | 22.40565 |
| IND | 3.92E+11 | 2.68E+23 | 4.91E+11 | 20.20428 |
| ITA | 2.38E+11 | 8.38E+22 | 2.89E+11 | 12.75623 |
| JPN | 2.33E+11 | 9.52E+22 | 2.73E+11 | 5.349436 |
| KOR | 3.99E+10 | 2.05E+21 | 4.38E+10 | 2.967281 |
| MEX | 5.04E+10 | 3.47E+21 | 5.89E+10 | 4.517596 |
| RUS | 4.14E+11 | 2E+23 | 4.38E+11 | 31.53654 |
| SAU | 9.69E+10 | 1.22E+22 | 1.07E+11 | 15.40036 |
| TUR | 1.92E+11 | 5.1E+22 | 2.26E+11 | 24.81534 |
| USA | 7.46E+11 | 8.06E+23 | 8.67E+11 | 4.277468 |
| ZAF | 3.96E+10 | 1.75E+21 | 4.16E+10 | 12.1879 |

**Table SI 3** Emissions kt model evaluation (avg)

| **Country** | **MAE** | **MSE** | **RMSE** | **MAPE** |
| --- | --- | --- | --- | --- |
| ARG | 35014.82 | 1.45E+09 | 36856.74 | 20.2654 |
| AUS | 32574.44 | 1.74E+09 | 37673.54 | 8.490102 |
| BRA | 68896.83 | 7.8E+09 | 81082.18 | 15.71501 |
| CAN | 83203.96 | 1.27E+10 | 93876.25 | 15.00415 |
| CHN | 2668584 | 1.03E+13 | 2988651 | 29.91151 |
| DEU | 46375.35 | 3.22E+09 | 51449.1 | 6.180919 |
| ESP | 86445.7 | 1.13E+10 | 102832.7 | 33.07117 |
| FRA | 41347.68 | 2.47E+09 | 49131.33 | 12.76817 |
| GBR | 74832.03 | 9.02E+09 | 94011.32 | 18.32786 |
| IDN | 73906.14 | 9.82E+09 | 86345.15 | 15.90889 |
| IND | 541946.9 | 4.13E+11 | 634339.5 | 27.74095 |
| ITA | 134197.9 | 3.09E+10 | 160664.2 | 38.07403 |
| JPN | 53859.78 | 5.3E+09 | 68888.19 | 4.652432 |
| KOR | 73013.85 | 7.52E+09 | 83275.59 | 12.4621 |
| MEX | 58830.28 | 6.21E+09 | 71097.24 | 12.64898 |
| RUS | 228570.1 | 1.12E+11 | 247783.1 | 14.03615 |
| SAU | 61720.32 | 5.85E+09 | 72592.66 | 11.83987 |
| TUR | 52260.67 | 5.29E+09 | 61373.01 | 15.37904 |
| USA | 735085.2 | 8.8E+11 | 858891.2 | 14.51849 |
| ZAF | 59775.44 | 7.04E+09 | 69075.82 | 14.13438 |

**Table SI 4** Biocapacity forecast model evaluation (avg)

| **Country** | **MAE** | **MSE** | **RMSE** | **MAPE** |
| --- | --- | --- | --- | --- |
| ARG | 16781805 | 3.8E+14 | 19221914 | 6.056389 |
| AUS | 20292373 | 5.47E+14 | 22072005 | 6.959919 |
| BRA | 39053961 | 2.04E+15 | 41330164 | 2.164241 |
| CAN | 5477361 | 5.59E+13 | 7445900 | 1.008191 |
| CHN | 68246690 | 8.55E+15 | 74007951 | 5.341585 |
| DEU | 8207774 | 1.18E+14 | 9929196 | 5.986129 |
| ESP | 5983173 | 5.82E+13 | 7584039 | 10.22849 |
| FRA | 11843427 | 2.53E+14 | 14785989 | 7.413067 |
| GBR | 5615390 | 4.44E+13 | 6239404 | 7.911329 |
| IDN | 18857311 | 4.57E+14 | 21118130 | 5.922857 |
| IND | 40406597 | 2.92E+15 | 47242605 | 7.422962 |
| ITA | 4368508 | 2.86E+13 | 5027551 | 8.056754 |
| JPN | 1503896 | 3.99E+12 | 1688110 | 1.964215 |
| KOR | 1098877 | 1.95E+12 | 1232968 | 3.287568 |
| MEX | 3509170 | 1.84E+13 | 4197355 | 2.444831 |
| RUS | 78095767 | 1.5E+16 | 85173979 | 8.06526 |
| SAU | 2460888 | 8.25E+12 | 2501569 | 18.05744 |
| TUR | 4464788 | 3.78E+13 | 5385549 | 4.211634 |
| USA | 24954066 | 9.7E+14 | 30366220 | 2.25621 |
| ZAF | 1408771 | 3.21E+12 | 1790875 | 2.545561 |

**Table SI 5** Forest km2 forecast model evaluation (avg)

| **Country** | **MAE** | **MSE** | **RMSE** | **MAPE** |
| --- | --- | --- | --- | --- |
| ARG | 10778.71 | 1.93E+08 | 12421.47 | 3.638236 |
| AUS | 18519.35 | 7.62E+08 | 26584.93 | 1.39289 |
| BRA | 122441.3 | 3.09E+10 | 148466 | 2.416103 |
| CAN | 974.4529 | 2881662 | 1135.514 | 0.028067 |
| CHN | 68464.82 | 1.36E+10 | 77715.67 | 3.318626 |
| DEU | 79.88221 | 14938.96 | 116.8389 | 0.069974 |
| ESP | 7337.192 | 1.34E+08 | 8925.062 | 3.970132 |
| FRA | 2693.721 | 16214798 | 3057.26 | 1.618021 |
| GBR | 331.8324 | 196213.5 | 409.4255 | 1.062938 |
| IDN | 44224.6 | 4.37E+09 | 50434.85 | 4.575942 |
| IND | 7207.282 | 84782756 | 8331.893 | 1.023745 |
| ITA | 1642.29 | 6114956 | 1943.94 | 1.781965 |
| JPN | 528.8228 | 359139.4 | 558.1621 | 0.211974 |
| KOR | 131.5325 | 40267.6 | 167.1052 | 0.207343 |
| MEX | 5795.766 | 65634252 | 6573.126 | 0.870939 |
| RUS | 23853.63 | 7.73E+08 | 27251.92 | 0.292777 |
| SAU | 3256.667 | 31817633 | 3256.667 | 33.33333 |
| TUR | 3677.229 | 25925450 | 4182.568 | 1.717122 |
| USA | 21036.2 | 6.79E+08 | 23958.73 | 0.680509 |
| ZAF | 829.7965 | 2750595 | 957.5667 | 0.480034 |

**Table SI 6** Crops km2 forecast model evaluation (avg)

| **Country** | **MAE** | **MSE** | **RMSE** | **MAPE** |
| --- | --- | --- | --- | --- |
| ARG | 74272.92 | 1.03E+10 | 101409.4 | 6.399818 |
| AUS | 431252.9 | 2.48E+11 | 460069.7 | 11.49832 |
| BRA | 35368.94 | 1.96E+09 | 43736.47 | 1.507069 |
| CAN | 30875 | 1.16E+09 | 34076.62 | 5.296801 |
| CHN | 183281.4 | 6.45E+10 | 209891.6 | 3.467591 |
| DEU | 1856.515 | 5093377 | 2135.767 | 1.108774 |
| ESP | 14875.79 | 2.91E+08 | 16901.16 | 5.538999 |
| FRA | 1720.949 | 7928938 | 2147.532 | 0.597292 |
| GBR | 9462.482 | 1.07E+08 | 10165.57 | 5.445724 |
| IDN | 44685.94 | 2.69E+09 | 50677.62 | 7.743302 |
| IND | 9035.099 | 1.83E+08 | 10341.97 | 0.503079 |
| ITA | 4037.566 | 28525900 | 4960.927 | 3.047331 |
| JPN | 2962.939 | 13322325 | 3417.982 | 6.556688 |
| KOR | 636.3225 | 749908.5 | 739.1109 | 3.650545 |
| MEX | 55403.92 | 4.6E+09 | 67369.06 | 5.586144 |
| RUS | 6216.273 | 44097379 | 6475.541 | 0.288428 |
| SAU | 96498.65 | 1.66E+10 | 107345.4 | 5.560871 |
| TUR | 23534.32 | 7.21E+08 | 26293.95 | 6.108484 |
| USA | 37442.95 | 2.28E+09 | 44675.88 | 0.920985 |
| ZAF | 11617.67 | 2.16E+08 | 12470.67 | 1.203058 |

**Table SI 7** kwh per capita forecast model evaluation (avg)

| **Country** | **MAE** | **MSE** | **RMSE** | **MAPE** |
| --- | --- | --- | --- | --- |
| ARG | 270.0973 | 101414.5 | 300.4861 | 9.298558 |
| AUS | 1713.389 | 4192279 | 2044.598 | 16.7191 |
| BRA | 242.6138 | 78252.69 | 271.5918 | 9.804152 |
| CAN | 1978.413 | 4871940 | 2193.778 | 12.6379 |
| CHN | 611.0419 | 828363.5 | 700.4752 | 18.7057 |
| DEU | 483.9238 | 461066.4 | 587.4315 | 6.819861 |
| ESP | 1261.915 | 2522810 | 1538.477 | 23.11571 |
| FRA | 1249.926 | 2281004 | 1502.375 | 17.55592 |
| GBR | 1125.37 | 1795815 | 1326.832 | 21.1334 |
| IDN | 150.3793 | 39117.32 | 193.5405 | 18.05032 |
| IND | 145.1393 | 28273.56 | 167.2905 | 20.01761 |
| ITA | 1176.491 | 2031239 | 1424.061 | 22.79825 |
| JPN | 1188.452 | 2495636 | 1384.319 | 14.89794 |
| KOR | 443.7615 | 426285.9 | 639.7247 | 4.39803 |
| MEX | 394.4597 | 203358.4 | 450.3069 | 18.57843 |
| RUS | 1095.526 | 2020134 | 1164.096 | 16.97254 |
| SAU | 621.303 | 504060 | 709.103 | 6.963336 |
| TUR | 269.9603 | 87078.79 | 293.1343 | 10.39802 |
| USA | 918.8591 | 1575864 | 1075.627 | 7.017759 |
| ZAF | 531.5347 | 464945.8 | 636.0471 | 12.35253 |

**Table SI 8** kg oileq PerCapita mode forecast evaluation (avg)

| **Country** | **MAE** | **MSE** | **RMSE** | **MAPE** |
| --- | --- | --- | --- | --- |
| ARG | 292.5867 | 96635.41 | 305.5181 | 14.98337 |
| AUS | 440.2707 | 320799.1 | 565.2536 | 7.994048 |
| BRA | 211.1651 | 57286.31 | 236.2993 | 14.93941 |
| CAN | 841.0628 | 1043916 | 952.1741 | 10.81457 |
| CHN | 416.347 | 280951.1 | 471.7929 | 20.69262 |
| DEU | 634.718 | 674179.5 | 684.6788 | 16.34578 |
| ESP | 811.4754 | 922377.2 | 958.7331 | 30.95807 |
| FRA | 739.3569 | 727020.8 | 848.998 | 19.47835 |
| GBR | 639.5819 | 536602.6 | 730.0147 | 21.82935 |
| IDN | 52.77493 | 5555.606 | 61.4759 | 6.162887 |
| IND | 96.08415 | 12130.54 | 109.5353 | 16.13079 |
| ITA | 727.0828 | 771409.1 | 876.1951 | 28.03629 |
| JPN | 640.8865 | 664544.6 | 746.8917 | 18.06773 |
| KOR | 385.3901 | 300775.9 | 435.2088 | 7.594349 |
| MEX | 140.7941 | 30720.36 | 167.8523 | 9.000479 |
| RUS | 702.283 | 1223053 | 761.4577 | 14.32328 |
| SAU | 765.7296 | 650834.5 | 801.2073 | 11.30332 |
| TUR | 154.7476 | 29238.84 | 170.0891 | 10.02815 |
| USA | 608.7574 | 510632.1 | 710.9019 | 8.765712 |
| ZAF | 227.9442 | 59804.02 | 243.6388 | 8.293209 |

**Table SI 9** emissions Mt cap model forecast evaluation (avg)

| **Country** | **MAE** | **MSE** | **RMSE** | **MAPE** |
| --- | --- | --- | --- | --- |
| ARG | 0.56598 | 0.370016 | 0.595643 | 13.67669 |
| AUS | 1.71564 | 5.909087 | 2.053161 | 10.45643 |
| BRA | 0.365326 | 0.205026 | 0.417159 | 16.86498 |
| CAN | 2.191616 | 6.946064 | 2.48673 | 13.88669 |
| CHN | 1.390401 | 2.932903 | 1.500975 | 21.62438 |
| DEU | 0.524461 | 0.363958 | 0.598965 | 5.721206 |
| ESP | 2.700905 | 10.52071 | 3.195005 | 48.02727 |
| FRA | 0.871181 | 0.999094 | 0.99951 | 17.66015 |
| GBR | 1.532601 | 3.647797 | 1.880471 | 24.16474 |
| IDN | 0.148249 | 0.03214 | 0.172089 | 8.29173 |
| IND | 0.389481 | 0.21209 | 0.453339 | 25.51947 |
| ITA | 1.929644 | 5.595166 | 2.334722 | 33.06779 |
| JPN | 0.354508 | 0.22347 | 0.46114 | 3.906747 |
| KOR | 1.172978 | 1.88885 | 1.334065 | 10.10056 |
| MEX | 0.250608 | 0.118558 | 0.32764 | 6.462085 |
| RUS | 1.263663 | 4.003984 | 1.349199 | 11.16243 |
| SAU | 0.936857 | 1.898398 | 1.347011 | 5.756664 |
| TUR | 0.750878 | 0.756945 | 0.840225 | 16.91881 |
| USA | 2.88984 | 11.40402 | 3.347428 | 18.08659 |
| ZAF | 0.650923 | 0.635764 | 0.728615 | 8.07946 |

**Table SI 10** gdp PerCap model forecast evaluation (avg)

| **Country** | **MAE** | **MSE** | **RMSE** | **MAPE** |
| --- | --- | --- | --- | --- |
| ARG | 2983.318 | 10174182 | 3149.41 | 22.37996 |
| AUS | 1578.737 | 4674409 | 1877.075 | 2.862667 |
| BRA | 1112.46 | 1591876 | 1229.064 | 12.87501 |
| CAN | 3946.266 | 20283376 | 4143.268 | 9.324776 |
| CHN | 1702.085 | 4393298 | 2029.743 | 24.15372 |
| DEU | 777.6934 | 936285.7 | 951.4828 | 1.999642 |
| ESP | 2432.271 | 10948282 | 2958.83 | 9.450114 |
| FRA | 2091.73 | 6493607 | 2499.576 | 5.712304 |
| GBR | 3243.404 | 15782063 | 3833.878 | 7.304572 |
| IDN | 594.3487 | 535029.3 | 710.7361 | 18.89679 |
| IND | 284.4815 | 120164.7 | 346.5037 | 19.04857 |
| ITA | 5298.011 | 39554899 | 6287.835 | 17.02252 |
| JPN | 1371.252 | 3712221 | 1651.291 | 4.02378 |
| KOR | 553.827 | 404844.9 | 608.045 | 2.153966 |
| MEX | 419.9368 | 285220 | 481.2367 | 4.48157 |
| RUS | 2330.61 | 7270922 | 2473.059 | 25.65392 |
| SAU | 2187.131 | 6682118 | 2355.195 | 10.89333 |
| TUR | 2130.458 | 5845411 | 2410.354 | 21.16713 |
| USA | 2372.185 | 8144703 | 2660.958 | 4.302267 |
| ZAF | 1076.295 | 1328664 | 1118.877 | 17.5626 |

**Table SI 11** Area PerCap model forecast evaluation (avg)

| **Country** | **MAE** | **MSE** | **RMSE** | **MAPE** |
| --- | --- | --- | --- | --- |
| ARG | 0.266441 | 0.119093 | 0.312641 | 4.306824 |
| AUS | 3.054803 | 10.94123 | 3.190699 | 9.690558 |
| BRA | 0.232312 | 0.080843 | 0.278234 | 5.388217 |
| CAN | 0.377031 | 0.414347 | 0.469666 | 1.7575 |
| CHN | 0.01518 | 0.000399 | 0.018078 | 1.947324 |
| DEU | 0.004787 | 3.76E-05 | 0.00584 | 0.596873 |
| ESP | 0.049893 | 0.00409 | 0.060111 | 4.335641 |
| FRA | 0.008564 | 0.000133 | 0.010718 | 0.73032 |
| GBR | 0.038723 | 0.001943 | 0.043622 | 3.302896 |
| IDN | 0.075827 | 0.01013 | 0.09004 | 5.542115 |
| IND | 0.011874 | 0.000269 | 0.014347 | 2.770786 |
| ITA | 0.029063 | 0.001216 | 0.034763 | 3.48724 |
| JPN | 0.012868 | 0.000309 | 0.015239 | 1.66366 |
| KOR | 0.024453 | 0.001212 | 0.030066 | 2.719471 |
| MEX | 0.088577 | 0.019855 | 0.113174 | 4.863691 |
| RUS | 0.70383 | 1.453852 | 0.775188 | 6.661927 |
| SAU | 1.404476 | 2.779196 | 1.549833 | 23.55818 |
| TUR | 0.032484 | 0.001883 | 0.040117 | 3.360805 |
| USA | 0.082092 | 0.015379 | 0.093456 | 2.590896 |
| ZAF | 0.108704 | 0.032751 | 0.135055 | 4.746578 |

**Table SI 11** Biocap PerCap model forecast evaluation (avg)

| **Country** | **MAE** | **MSE** | **RMSE** | **MAPE** |
| --- | --- | --- | --- | --- |
| ARG | 0.336924 | 0.210302 | 0.421903 | 5.198063 |
| AUS | 0.936154 | 1.396579 | 1.042241 | 7.250171 |
| BRA | 0.548588 | 0.511047 | 0.644475 | 6.138892 |
| CAN | 0.903006 | 1.29764 | 1.003209 | 5.873214 |
| CHN | 0.052703 | 0.003657 | 0.056405 | 5.344412 |
| DEU | 0.101649 | 0.018948 | 0.134224 | 6.189391 |
| ESP | 0.21666 | 0.062846 | 0.25002 | 16.86271 |
| FRA | 0.24612 | 0.094867 | 0.291286 | 9.822518 |
| GBR | 0.171291 | 0.035418 | 0.185407 | 15.52927 |
| IDN | 0.104292 | 0.016053 | 0.121726 | 8.262826 |
| IND | 0.020049 | 0.000561 | 0.023681 | 2.932513 |
| ITA | 0.122329 | 0.020642 | 0.139492 | 12.91344 |
| JPN | 0.027759 | 0.00109 | 0.032269 | 3.388174 |
| KOR | 0.054536 | 0.004165 | 0.059979 | 5.85061 |
| MEX | 0.033849 | 0.001677 | 0.039544 | 2.741282 |
| RUS | 0.407942 | 0.390947 | 0.461208 | 6.093234 |
| SAU | 0.126754 | 0.017309 | 0.13138 | 17.80153 |
| TUR | 0.095161 | 0.018813 | 0.11074 | 6.737472 |
| USA | 0.112079 | 0.01877 | 0.134628 | 3.205854 |
| ZAF | 0.164362 | 0.038273 | 0.185773 | 15.65808 |

**Table SI 13** EFConsPerCap model forecast evaluation (avg)

| **Country** | **MAE** | **MSE** | **RMSE** | **MAPE** |
| --- | --- | --- | --- | --- |
| ARG | 0.27966 | 0.144799 | 0.343381 | 8.693426 |
| AUS | 0.765605 | 0.722489 | 0.849859 | 9.95888 |
| BRA | 0.169801 | 0.038797 | 0.195433 | 6.317036 |
| CAN | 0.644657 | 0.547613 | 0.727508 | 7.768937 |
| CHN | 0.814701 | 0.778624 | 0.881653 | 24.64615 |
| DEU | 0.29005 | 0.121328 | 0.347802 | 5.692444 |
| ESP | 1.66939 | 3.913805 | 1.974857 | 40.54746 |
| FRA | 0.347097 | 0.175956 | 0.419292 | 7.177283 |
| GBR | 0.742963 | 0.826709 | 0.888621 | 15.80516 |
| IDN | 0.119983 | 0.025668 | 0.146097 | 7.55621 |
| IND | 0.170878 | 0.038957 | 0.195077 | 15.52317 |
| ITA | 1.087704 | 1.853602 | 1.33298 | 24.42007 |
| JPN | 0.680521 | 0.701765 | 0.733131 | 14.61074 |
| KOR | 0.538543 | 0.444279 | 0.635386 | 9.266168 |
| MEX | 0.46274 | 0.340981 | 0.560571 | 18.04725 |
| RUS | 0.691067 | 0.778604 | 0.760976 | 12.8545 |
| SAU | 1.11585 | 1.635901 | 1.165349 | 19.92652 |
| TUR | 0.332104 | 0.144421 | 0.359263 | 10.29118 |
| USA | 1.122671 | 1.634329 | 1.276114 | 13.54853 |
| ZAF | 0.583961 | 0.527069 | 0.69722 | 14.91111 |

|  |
| --- |

**Figure SI 24** Environmental Policy Stringency (EPS) index

This chart shows the Environmental Policy Stringency Index (EPS) used by the OECD, which serves as a nuanced, country-specific gauge designed to assess and compare the rigor of environmental regulations across nations on an international scale. This index quantifies stringency by evaluating the extent to which environmental policies impose a direct or indirect cost on pollution or behaviours detrimental to the environment, thereby offering valuable insights into how different nations are tackling environmental challenges through policy measures. The EPS index stands as a critical tool for policymakers, researchers, and environmental advocates seeking to understand and enhance the effectiveness of environmental regulations globally^1^.

| 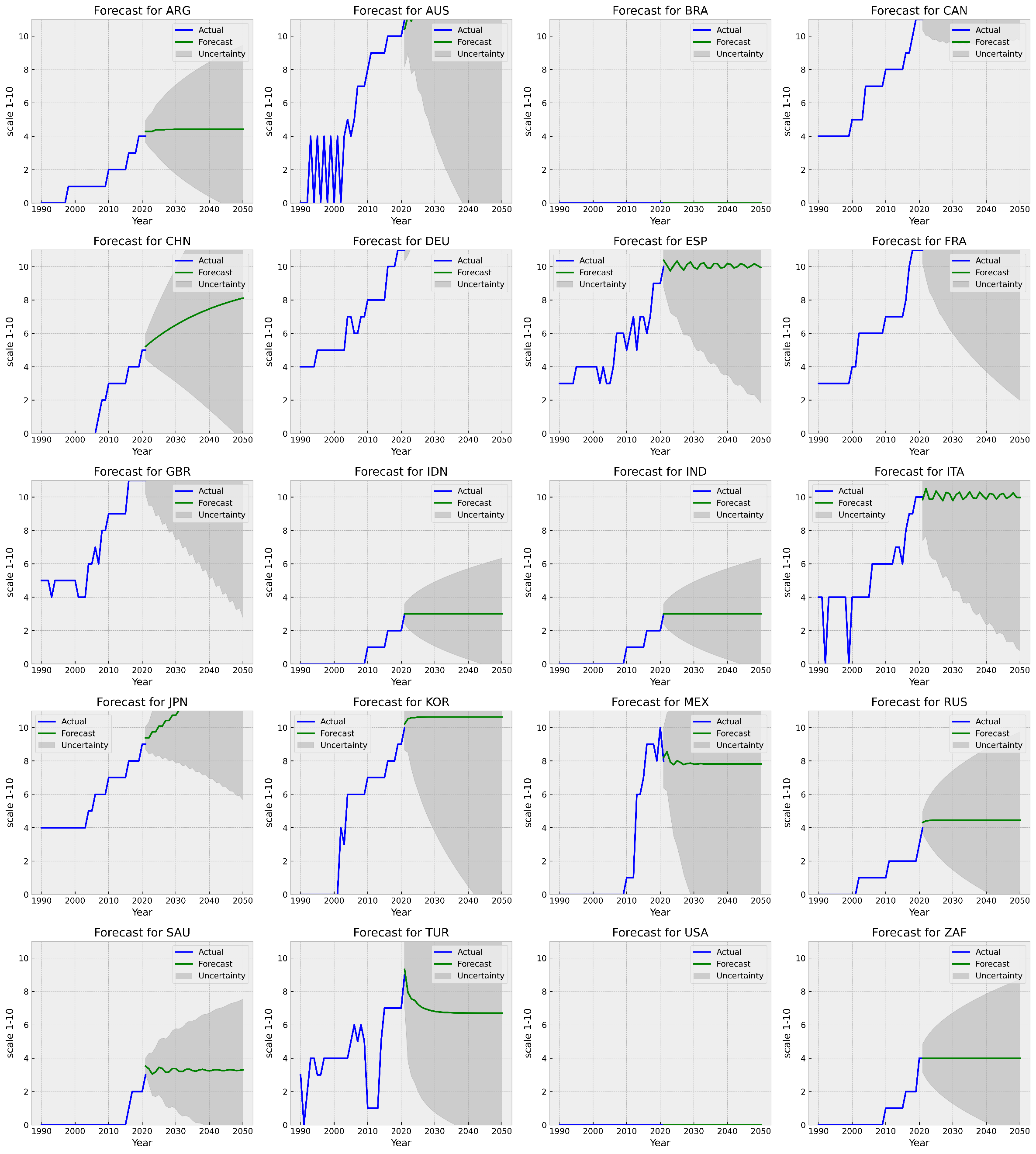 |
| --- |

**Figure SI 25** Cross Sectorial Environmental Policies for climate change

This graph shows our own forecast of cross sectorial policies for climate change mitigation in the G20 countries, this delineates the extent to which enacted policies foster a reduction in emissions, thereby incentivizing more environmentally responsible actions. Developed by the International Programme for Action on Climate, in scale from 1–10 examines over 130 policy variables, which are grouped into 56 major climate actions and policies. The database calculates both individual and average stringency scores for policies, as well as the number of policies enacted in various sectors. This framework aims to provide a clear, quantifiable way to understand how different countries are addressing climate change through their policies, making it a valuable tool for policymakers, researchers, and environmental advocates looking to analyse and compare climate action efforts globally^2,3^.

| 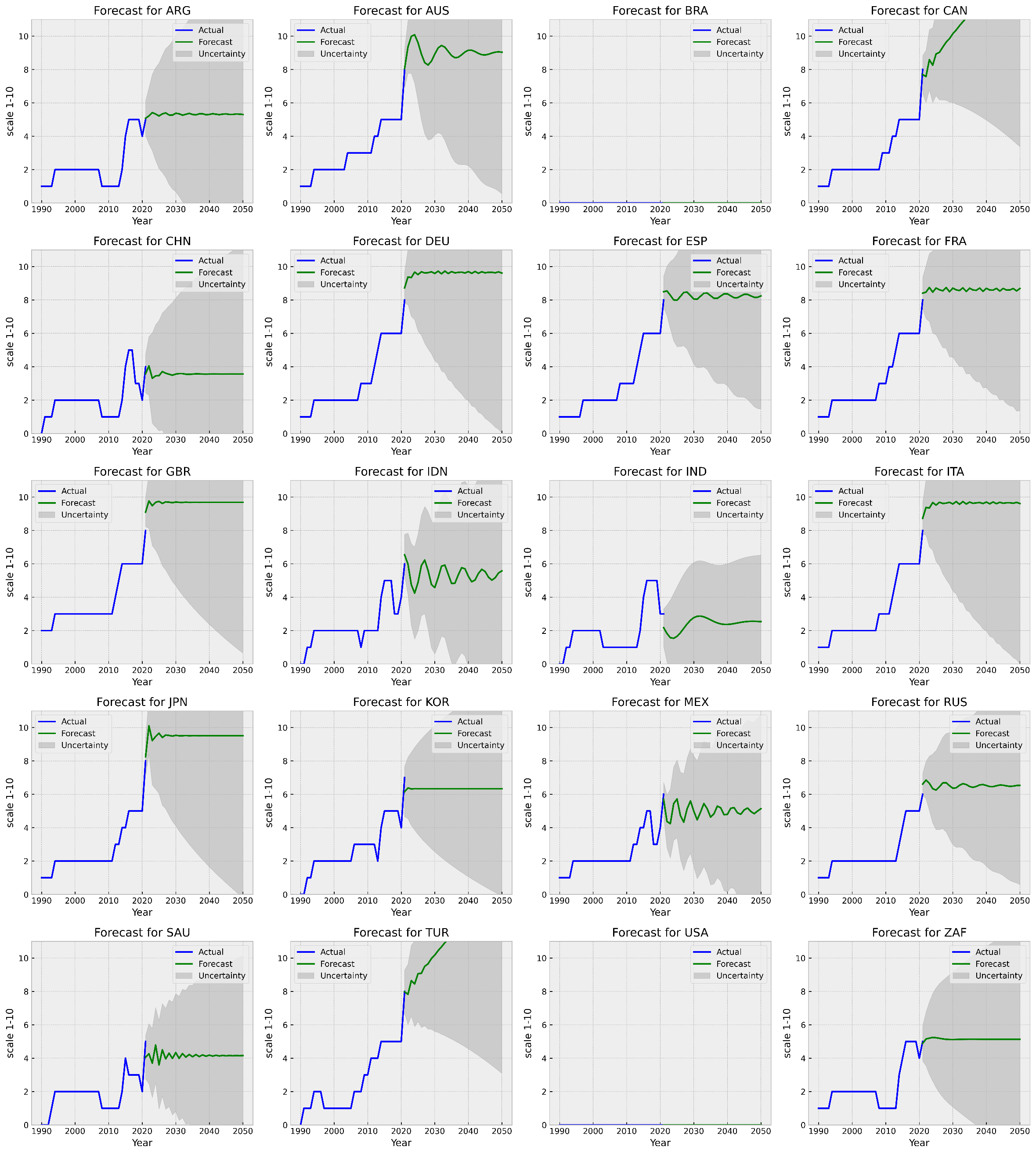 |
| --- |

**Figure SI 26** International Environmental Policies for climate change

This graph shows our own forecast of International Environmental policies for climate change mitigation in the G20 countries, this delineates the extent to which enacted policies foster a reduction in emissions, thereby incentivizing more environmentally responsible actions. Developed by the International Programme for Action on Climate, in scale from 1–10 examines over 130 policy variables, which are grouped into 56 major climate actions and policies. The database calculates both individual and average stringency scores for policies, as well as the number of policies enacted in various sectors. This framework aims to provide a clear, quantifiable way to understand how different countries are addressing climate change through their policies, making it a valuable tool for policymakers, researchers, and environmental advocates looking to analyse and compare climate action efforts globally^2,3^.

| 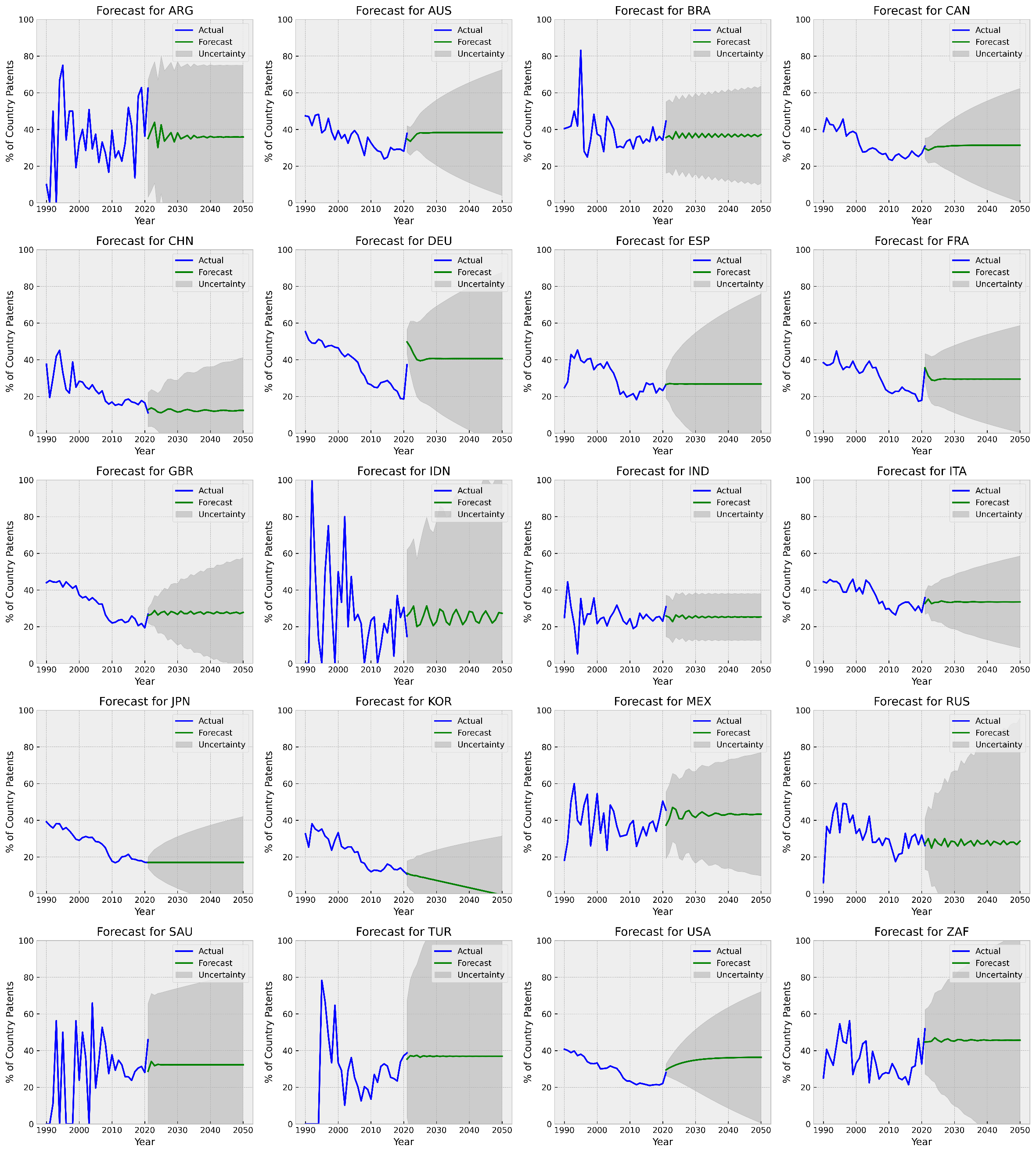 |
| --- |

**Figure SI 27** Development of Technologies for Environmental Management

This graph shows a forecast of innovation in environment-related technologies, the units are percentage of country patents. Patent statistics and indicators play a crucial role in monitoring advancements in environmentally friendly technologies. They provide a framework for evaluating the innovation capabilities of various countries and aid in shaping governmental policies related to the environment and innovation. This forecast highlights the number of new inventions in eco-friendly technologies, pinpointing the inventors' countries and the distribution of inventions^4^.

| 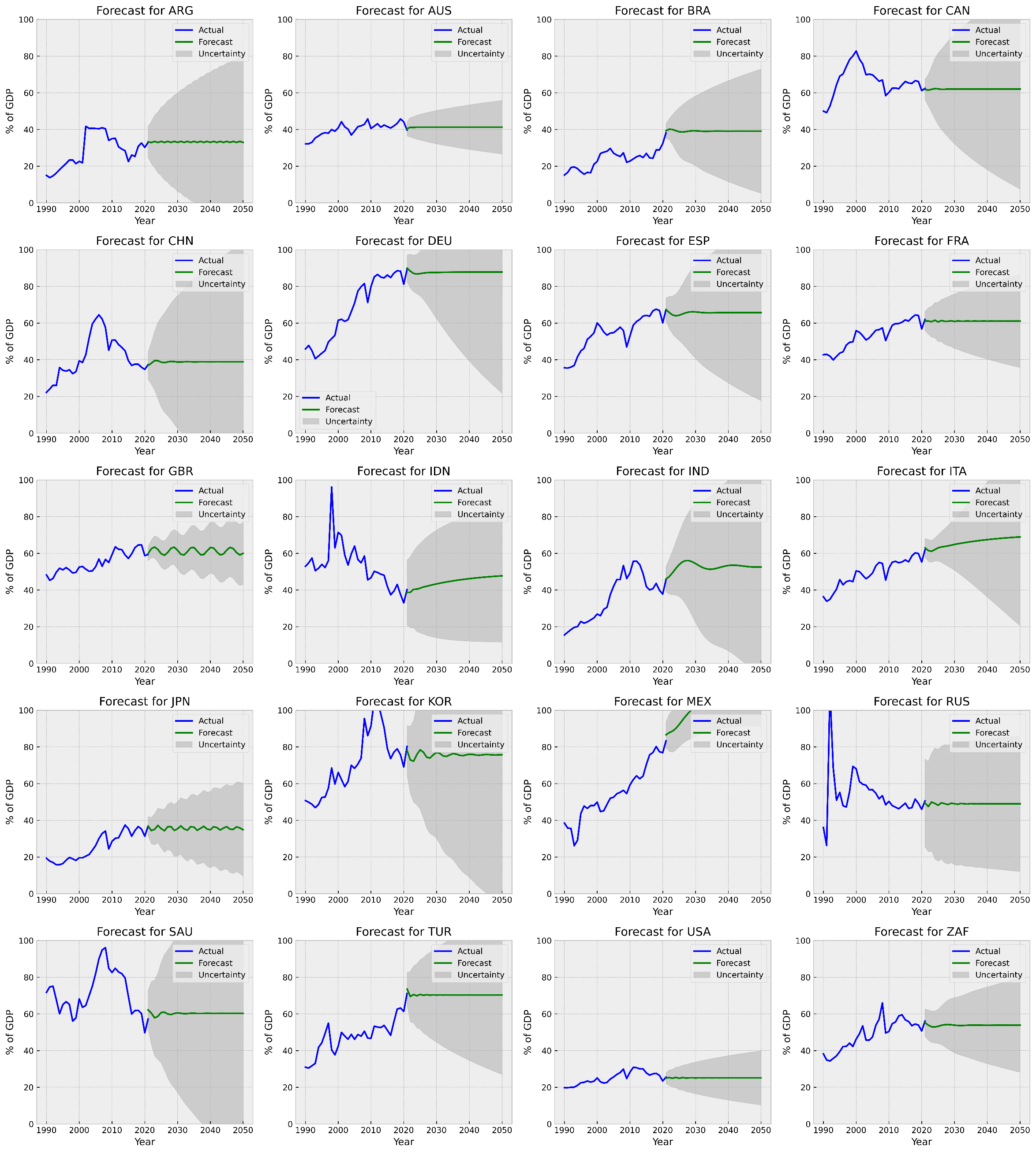 |
| --- |

**Figure SI 28** International Trade

This graph present both retrospective and prospective views on how trade relates to GDP in different national economies, with varying degrees of uncertainty and trends over the forecast period. Trade is displayed as a percentage of GDP, the blue line shows the historical trend, while the green line indicates the model's forecast from around 2021 onward, extending to 2050, The shaded grey area shows the uncertainty associated with the forecast. This sort of analysis can be vital for policymakers, economists, and investors who are interested in understanding potential future economic conditions^5^.

1 Kruse, T., Dechezleprêtre, A., Saffar, R. & Robert, L. Measuring environmental policy stringency in OECD countries. (2022). <https://doi.org:doi:https://doi.org/10.1787/90ab82e8-en>

2 Nachtigall, D., Lutz, L., Rodríguez, M. C., Haščič, I. & Pizarro, R. The climate actions and policies measurement framework. (2022). <https://doi.org:doi:https://doi.org/10.1787/2caa60ce-en>

3 OECD. *Environment at a Glance*. (2006).

4 OECD. *Patents on environment technologies (indicator), Accessed on 08 March 2024*, 2024).

5 Bank, T. W. (2024).
